# Complexity and Specificity of the Neutrophil Transcriptomes in Juvenile Idiopathic Arthritis

**DOI:** 10.1038/srep27453

**Published:** 2016-06-07

**Authors:** Zihua Hu, Kaiyu Jiang, Mark Barton Frank, Yanmin Chen, James N. Jarvis

**Affiliations:** 1 Center for Computational Research, New York State Center of Excellence in Bioinformatics & Life Sciences, State University of New York at Buffalo, Buffalo, NY 14260, USA.; 2 Department of Ophthalmology, Department of Biostatistics, Department of Medicine, State University of New York at Buffalo, Buffalo, NY 14260, USA; 3 SUNY Eye Institute, Buffalo, NY 14260, USA; 4 Department of Pediatrics, Division of Allergy/Immunology/Rheumatology, University at Buffalo, Buffalo, NY 14203, USA; 5 Arthritis & Immunology Program, Oklahoma Medical Research Foundation, Oklahoma City, OK, USA; 6 Graduate Program in Genetics, Genomics, & Bioinformatics, University at Buffalo, Buffalo, NY 14203, USA

## Abstract

NIH projects such as ENCODE and Roadmap Epigenomics have revealed surprising complexity in the transcriptomes of mammalian cells. In this study, we explored transcriptional complexity in human neutrophils, cells generally regarded as nonspecific in their functions and responses. We studied distinct human disease phenotypes and found that, at the gene, gene isoform, and miRNA level, neutrophils exhibit considerable specificity in their transcriptomes. Thus, even cells whose responses are considered non-specific show tailoring of their transcriptional repertoire toward specific physiologic or pathologic contexts. We also found that miRNAs had a global impact on neutrophil transcriptome and are associated with innate immunity in juvenile idiopathic arthritis (JIA). These findings have important implications for our understanding of the link between genes, non-coding transcripts and disease phenotypes.

The completion of the human genome assembly more than 10 years ago left a great deal of work still to be done. Although the genome sequence has provided some insights into how genetic variation may lead to differences in phenotypes, including disease phenotypes, there still remains much to be learned about the vast regions of the genome that do not encode annotated genes. This has become increasingly important as we have come to recognize that most genetic risk for complex traits, for example, juvenile idiopathic arthritis (JIA), resides outside of the protein-coding parts of the genome^1^,^2^. Thus, additional efforts were put forward through NIH’s Encyclopedia of DNA Elements (ENCODE) and Roadmap Epigenomics projects to define the functional elements within the genome, including those that are far (in genomic terms) from annotated genes.

Among the most interesting findings to emerge from the ENCODE and Roadmap Epigenomics projects has been the wealth of data about the complexity of the transcriptomes of both simple organisms and mammalian cells. The human transcriptome is far more complicated than was initially anticipated from the structure of the human genome^3^,^4^. While some controversy still exists regarding just how much of the human genome is transcribed^5^, it is now clear that a large number of RNA transcripts do not originate from protein-coding regions and that many of these non-coding transcripts are functional^6^. Some of the most studied non-coding transcripts are miRNAs, which mediate gene regulation post-transcriptionally and have the potential to silence gene expression via multiple mechanisms^7^. The complexity of the transcriptome is highlighted further by the extensive use of alternative splicing of RNA transcripts, now estimated to occur in 40–60% of mammalian genes^8^. Early work from the ENCODE consortium discovered that 90% of 399 protein-encoding genes examined had either a previously unannotated exon or alternative transcription start site^9^.

Juvenile idiopathic arthritis (JIA) is a complex human illness characterized by chronic inflammation and hypertrophy of synovial membranes. The polyarticular subtype of this disease shows both phenotypic and immunogenetic overlap with adult rheumatoid arthritis. We have previously reported that neutrophils from children with the polyarticular form of JIA exhibit specific transcriptional abnormalities that do not return to normal when the disease becomes inactive^10^. Furthermore, these transcriptional abnormalities are linked to aberrations in specific neutrophil metabolic functions^10^. Thus, although it is conventionally thought of as an autoimmune disease driven by disordered T cell responses^11^, increasing evidence points to an important role for innate immunity in the pathogenesis of JIA^10^,^12^,^13^. Furthermore, there is a growing body of data demonstrating the importance of neutrophils in shaping adaptive immune response^14^,^15^.

The studies we report here were undertaken to assess the abnormalities in JIA neutrophils by surveying the transcriptome in greater detail. Furthermore, we aimed to determine the specificity of JIA transcriptional abnormalities by comparing JIA neutrophils with those from another disease characterized by chronic inflammation of no-autoimmune origin in soft tissues, cystic fibrosis (CF). We have found that, at the gene, gene isoform, miRNA, and integrated miRNA-mRNA regulatory network levels, neutrophils exhibit considerable specificity in their transcriptomes.

## Results

### Exon and miRNA arrays reveal the complexity and specificity of the JIA neutrophil transcriptome

Exon and miRNA hybridization-based microarrays were used to generate mRNA, gene isoform, and miRNA expression profiles for 3 phenotypes. These phenotypes included 35 children with newly-diagnosed, untreated polyarticular, RF-negative JIA^16^, 15 children with cystic fibrosis (CF), and 43 healthy controls (HC). Children with CF were stable at the time they were studied and had no clinical evidence of worsening lung function. For gene expression studies, 216 and 5965 differentially expressed genes (*p* < = 0.001 and fold change > = 1.5) were selected between JIA and HC (Table S1 in Supplementary information) as well as between CF and HC (data not shown), respectively. The specificity of the JIA transcriptome was assessed by comparing children with JIA to children with CF. Significant differences were observed between the transcriptomes of neutrophils from the 2 diseases, as evidenced by the number of differentially expressed genes from JIA and CF when each was compared to HC. We next compared the 216 differentially expressed genes from JIA with the 5965 differentially expressed genes from the CF-HC comparison and found significant overlap. Specifically, 148 (68.52%) of differentially expressed genes from JIA were common to those from CF (*p* = 7.5E-28), while 68 (31.5%) differentially expressed genes were unique to JIA (Table S1 in Supplementary information). Thus, although we detected 216 neutrophil genes whose expression levels differed between children with untreated JIA and healthy controls, all but 68 of those genes also showed differential expression when children with CF were compared with HC, suggesting that the other 148 genes are part of the physiologic adaptation to chronic inflammation in soft tissues rather than disease-specific for JIA.

**Figure 1:**
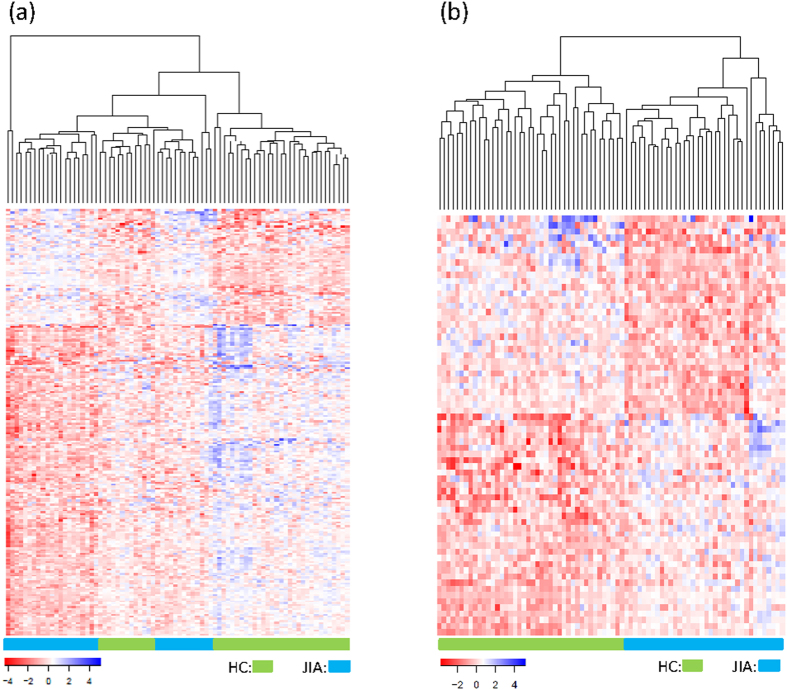
Unsupervised hierchical clustering analysis of gene expression for differentially expressed genes from 35 JIA and 43 HC samples. (**a**) The heatmap shows the median-normalized expression of individual genes across all samples for the 216 differentially expressed genes; (**b**) The heatmap shows the median-normalized expression of individual genes across all samples for the 68 differentially expressed genes unique to JIA, when compared to CF vs HC. Heatmap colors represent relative mRNA expression as indicated in the color key.

Hierarchical cluster analysis using the expression data of the 216 genes from both JIA and HC separated the JIA and HC into 3 clusters when at least one cluster was set to have samples from the same phenotype (Fig. 1a). While the largest cluster had 32 samples from JIA and 13 samples from HC with 71% accuracy in predicting the JIA phenotype, the other 2 clusters had samples from either JIA or HC. When these 148 common genes to CF were filtered out, we found a distinct separation of JIA and HC samples on hierarchical cluster analysis, as shown in the heatmap (Fig. 1b).

Similar results were obtained from both isoform and miRNA expression data analysis. While 9 and 70 differentially expressed miRNAs (FDR < = 0.1) were detected in JIA neutrophils (Table 1) and CF (Table S2 in Supplementary information), respectively, compared with healthy controls. 2 of those miRNAs (miR-494, miR-551a) are common to both groups (*p* = 3E-02). Similarly, at the isoform level, we found isoforms from 295 unique genes (Table S3 in Supplementary information) in children with JIA that were not expressed in HC and 4496 in children with CF displaying differential exon splicing (data not shown). At the same time, 257 of the differentially expressed isoforms were seen in both JIA and CF (*p* = 1.4E-81).

### Functional annotation of differentially expressed genes and target genes of differentially expressed miRNAs

**Figure 2:**
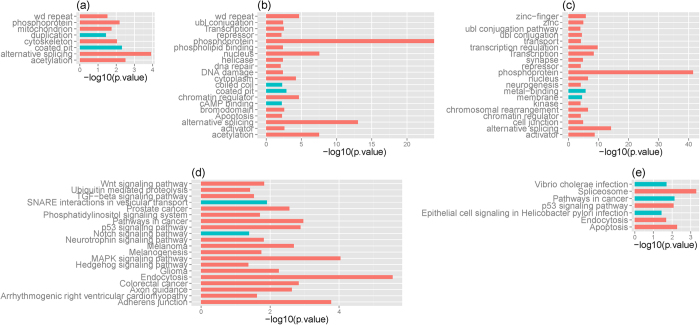
Functional annotation for differentially expressed genes, target genes of differentially expressed miRNAs, and isoforms in JIA. (**a**) *p* value of enriched SP-PIR functional categories from 216 differentially expressed genes; (**b**) *p* value of enriched SP-PIR functional categories from 296 isoforms; (**c**) *p* value of enriched SP-PIR functional categories from target genes of 9 differentially expressed miRNAs; (**d**) *p* value of enriched KEGG pathways from target genes of 9 differentially expressed miRNAs; (**e**) *p* value of enriched KEGG pathways from 296 isoforms. The enriched *p* value in each is depicted on axis x. Enriched function categories and pathways overlapping with those from CF are depicted as blue bars and those unique to JIA in orange bars.

Functional enrichment analyses of differentially expressed genes was undertaken using the Database for Annotation, Visualization and Integrated Discovery (DAVID) Software (v6.7)^17^. The analyses indicated that differentially expressed genes in children with JIA associated with limited number of biological functions, including alternative splicing, cytoskeleton, phosphoproteins, and wd repeat (Fig. 2a). The 296 isoforms differentially expressed in JIA and target genes of the 9 differentially expressed miRNAs were related to 42 and 67 biological functions (*p* < = 0.05), respectively. The top 20 functions with the lowest *p*-values are shown in Fig. 2(b,c). It is interesting that all three sets of genes (i.e., differentially expressed genes, isoforms, and miRNA target genes) were highly related to phosphoprotein function and alternative splicing processes. The latter consisted of 48%, 51.9%, and 60% total genes and had the lowest *p*-values of 10^−14^, 10^−4^, and 10^−13^ for miRNA target genes, differentially expressed genes, and isoforms, respectively, supporting the idea that JIA may be a disorder of transcriptional regulation and control^18^. Further enrichment analysis using the Kyoto Encyclopedia of Genes and Genomes (KEGG) indicated that the target genes of the 9 differentially expressed miRNAs seen in JIA were enriched in 22 pathways (*p* < 0.05). Figure 2d shows the top 20 pathways with the lowest *p*-values, the majority of which were involved in cell signaling, including the MAPK signaling pathway, which we have previously shown to be important in JIA neutrophils^12^,^19^. The 296 isoforms were also enriched in 7 pathways (*p* < 0.05) as shown in Fig. 2e, where three pathways involving endocytosis, p53 signaling pathway, and pathways in cancer were common to those seen in miRNA functional analysis.

Functional analysis of differentially expressed genes, isoforms, and miRNA target genes seen when children with JIA were compared to HC showed considerable overlap with those (compared with HC) seen in children with CF. The SP-PIR functional annotation demonstrated 62.5% (5/8), 90% (18/20), and 90% (18/20) overlap between JIA and CF for differentially expressed genes, isoforms, and miRNA target genes, respectively (Fig. 2a–c). Similar results were observed at the pathway level as shown in Fig. 2d,e, where 90% (18/20) and 57.1% (4/7) of enriched pathways display overlap between JIA and CF from target genes of differentially expressed miRNAs and differentially expressed isoforms, respectively.

### Correlation analysis of miRNA and mRNA expression reveals a global impact of miRNAs on the JIA neutrophil transcriptome

It is widely accepted that miRNAs mediate gene regulation by decreasing the stability of their target transcripts, leading to a reduced abundance of mRNA^7^. At the same time, miRNAs operate in complex networks, the structures of which are determined in part by the relative abundance of other competing mRNA targets for specific miRNAs^20^,^21^. In order to test whether miRNA-mediated regulation has a global effect on JIA neutrophil transcriptome, we computed the correlation between the expression of miRNAs and the expression of their target genes predicted by TargetScans^22^. If miRNAs have an impact on the JIA neutrophil transcriptome, more genes will display a negative correlation when compared to those from HC. To test this idea, we performed correlation analyses at three different levels. These included correlation between the expression of individual miRNAs and the expression of all target genes (which we denote as “miRNA level”), correlation between the expression of an individual gene and the expression of all miRNAs that target this gene (which we denote as “gene level”), and correlation between the expression of individual miRNA and the expression of individual gene (which we denote as “individual miRNA-mRNA level”).

**Figure 3:**
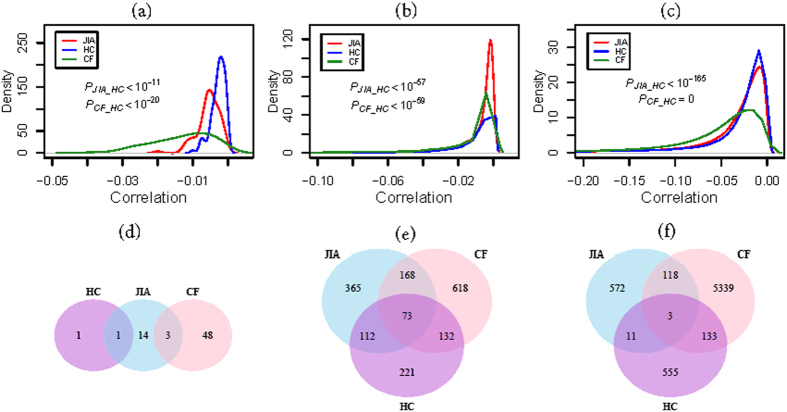
Correlation between miRNA expression and target mRNA expression. Distribution of Pearson correlation coefficients from (**a**) individual miRNAs to all target genes; (**b**) from individual genes to all miRNAs; and (**c**) from individual miRNAs to individual target genes. Both JIA and CF display significantly smaller negative correlation coefficients when compared to HC; (**d**) Venn diagram showing the number of shared and unique miRNAs whose expression is significantly anti-correlated with all target gene expression. Significant overlap was detected between JIA and HC (*p* = 0.005); (**e**) the number of shared and unique genes whose expression is significantly anti-correlated with all miRNAs. Significant overlaps were detected between JIA and CF (*p* = 5e-140), CF and HC (*p* = 9e-131), JIA and HC (*p* = 2e-135). (**f**) The number of shared and unique miRNA-mRNA pair with significant negative correlations (< = −0.3). Significant overlaps were detected between JIA and CF (*p* = 1.1e-60), CF and HC (*p* = 8.0e-75), and JIA and HC (*p* = 2.4e-08).

The distributions of Pearson negative correlation are shown in Fig. 3a–c, where both JIA and CF display significantly smaller negative correlations than those from HC (Wilcoxon signed-rank tests *p* from 0 to <10^−8^) in all three levels of analysis. Similar results were also obtained from Spearman’s rank negative correlation (data not shown). It is interesting to note that CF has the most significantly smaller negative correlations, consistent with the results from differentially expressed genes described above. To obtain significantly and inversely correlated miRNAs and mRNAs, permutation tests (see Material and Methods) were performed to estimate the *p*-values, which were used to establish *p*-value cutoffs. At the miRNA level we were able to identify 18 miRNAs from JIA, 51 from CF, and 2 from HC with significant correlations (*p* < 10^−3^; both Pearson *R* and Spearman’s rank *rho* < = −0.0075). While JIA and CF had far more significantly correlated miRNAs when compared to HC, a few of these 18 miRNAs from JIA were common between each other as shown in Fig. 3d, where 3 miRNAs overlap between JIA and CF and 1 between JIA and HC. At the gene level, 718 genes from JIA, 991 genes from CF, and 538 genes from HC were significantly and negatively correlated with miRNA expression (*p* < 10^−3^; both Pearson *R* and Spearman’s rank *rho* < = −0.02). In agreement with the results at the miRNA level, JIA and CF had more genes showing significant correlations than those from HC. The number of shared and unique mRNAs between and among JIA, CF, and HC is shown in Fig. 3e, with significant overlaps between all three phenotypes (*p* from 5 × 10^−140^ to 10^−130^). Finally, at the individual miRNA-mRNA level, we identified 704 miRNA-RefSeq gene pairs from JIA, 5993 miRNA-RefSeq gene pairs from CF, and 702 miRNA-RefSeq gene pairs from HC (*p* < 10^−3^; both Pearson *R* and Spearman’s rank *rho* < = −0.3). Consistent with the findings from the miRNA and the gene levels, CF had the largest number of miRNA-gene pairs which were significantly overlapped with those from JIA and HC (Fig. 3f). Taken together, these results suggest that miRNA-mediated gene regulation has a global impact on the JIA neutrophil transcriptome.

## Reconstruction of Integrated Regulatory Networks

In an effort to expand our study to a more complex and more physiologically relevant relationships between miRNAs and target genes, we utilized those significantly correlated individual miRNA-mRNA pairs from the above correlation analysis to reconstruct regulatory networks using Cytoscape^23^. To illustrate the interactions between miRNA target genes, we also incorporated protein-protein interactions^24^ to the network.

**Figure 4:**
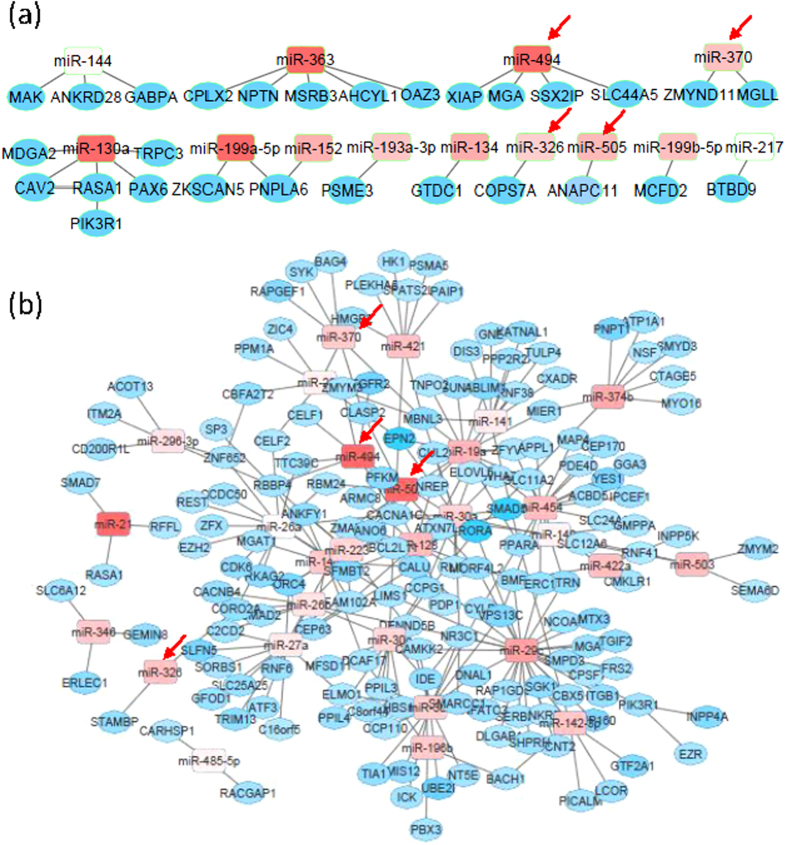
Integrated miRNA-gene regulatory networks from significantly up-regulated miRNAs and significantly down-regulated target genes. Graphical representation of regulatory networks for JIA (**a**) and CF (**b**). Rectangles: miRNAs. Circles: target genes. Red: up-regulated miRNAs and target genes when compared to HC. Blue: down-regulated miRNAs and target genes when compared to HC. Red arrows: common miRNAs between JIA and CF networks.

Regulatory networks from both JIA and CF displayed high degree of node connection (Figure S1 in Supplementary information). Since miRNAs reduce the abundance of their target transcripts, we next constructed regulatory networks for JIA and CF with miRNA and mRNA expression in the opposite directions (Figure S2 in Supplementary information), when compared to HC. Whereas in both expression situations JIA had many small networks, miRNA and gene nodes in CF were highly connected, forming large networks. It is important to note that significant overlaps between JIA and CF were observed for both genes (*p* < = 1E-59) and miRNAs (*p* < = 2.3E-03) in the regulatory networks (Table S4 in Supplementary information). To explore regulatory networks that might be directly involved in miRNA-mediated gene expression, we next focused more specifically on genes and miRNAs that had significant (*p* < = 0.05) expression differences between JIA and HC as well as between CF and HC. The constructed networks from significantly up-regulated miRNAs and significantly down-regulated target genes involved 13 miRNAs from JIA (Fig. 4a) and 29 miRNAs from CF (Fig. 4b), 4 (miR-494, miR-370, miR-326, and miR-505) of them were common (*p* = 1.2E-03, Table S4 in Supplementary information). Although networks constructed from significantly down-regulated miRNAs and significantly up-regulated target genes had few miRNA and mRNA nodes, significant overlaps were also observed between JIA and CF (Figure S3a,b and Table S4 in Supplementary information).

**Figure 5:**
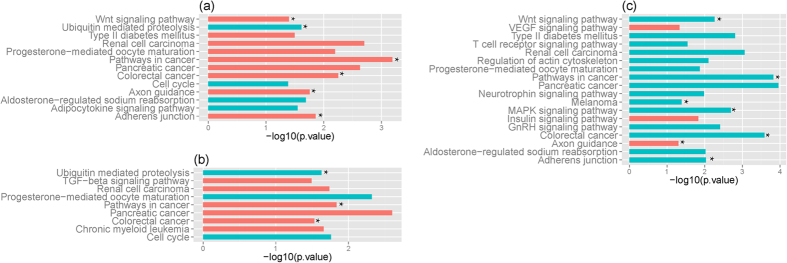
Functional annotation of genes in the integrated regulatory networks in JIA. (**a**) *p* value of enriched KEGG pathways from all genes of miRNA-gene regulatory network; (**b**) *p* value of enriched KEGG pathways from genes of the regulatory network constructed from up-regulated miRNAs and down-regulated target genes; (**c**) *p* value of enriched KEGG pathways from genes of the regulatory network constructed from hub miRNAs. The enriched *p* value in each is depicted on axis x. Enriched pathways overlapping with those from CF are depicted as blue bars and those unique to JIA in orange bars. Enriched pathways overlapping with those from target genes of the 9 differentially expressed miRNAs (Fig. 2d) are labeled with asterisks.

Functional annotation for genes in the networks indicated that majority of the enriched pathways from JIA were also enriched from genes seen in CF (Fig. 5a,b). It is worth noting that significant overlaps (*p* = 5.1E-06) were also observed between enriched pathways from network genes and target genes of differentially expressed miRNAs (Fig. 2). Those included wnt signalling pathways, ubiquitin mediated proteolysis, axon guidance, adherens junction, colorectal cancer, and pathway in cancer (Fig. 5a). Consistent with those from the target genes of differentially expressed miRNAs, the majority of the enriched pathways are related to cancer, including cell cycle, colorectal cancer, pathway in cancer, renal cell carcinoma, and pancreatic cancer. Supporting this result, a recent study indicated that children with JIA developed about four times as many new growths considered likely to be cancerous as children who did not have arthritis^25^.

It is known that network hubs are critical to the stability of the networks, and that miRNAs regulating multiple genes are likely to play important roles in network/biological function^26^,^27^. Selecting the top 5% miRNA nodes with the highest degree as hubs^28^, we observed 10 miRNA nodes with degrees between 10 and 30 for JIA and 12 miRNA nodes with degrees between 48 and 67 for CF (Figure S3a and S3b in Supplementary information). It is interesting to see that all hubs in either JIA or CF belong to different miRNA clusters; in this case, no overlap was observed between JIA and CF. On the other hand, 7 out of 18 (*p* = 6.1E-06) enriched KEGG pathways from genes of hub network in JIA (Fig. 5c) overlap with those from target genes of differentially expressed miRNAs (Fig. 2d).

### Functional studies reveal association between miRNAs and innate immunity in JIA

Although the classification of JIA is continuingly evolving and the disease is conventionally thought of as an autoimmune disease driven by disordered T cell responses, recent evidence points to an important role for innate immunity in the pathogenesis of JIA^10^,^12^,^13^ and the importance of neutrophils in shaping adaptive immune response^14^,^15^. To investigate the importance of miRNAs for innate immunity in JIA, we performed large scale analyses to study the functional associations of miRNA target genes with 1910 immunologic signatures (gene sets). This was done by computing the correlation of regulatory effect scores (RE-score)^29^ between individual immunologic signature and the target genes of individual miRNAs (see Material and Methods).

The distribution and frequency of RE-score correlations from JIA, CF, and HC is shown in Fig. 5a,b, where Pearson correlations from JIA vs HC and CF vs HC are depicted. While correlations from HC display bell shape distributions, those from JIA and CF are skewed to both ends. This finding is noted especially for the positive correlations. Wilcoxon signed-rank tests indicated that both JIA and CF had significantly larger positive correlations or significantly smaller negative correlations than those from HC (*p* = 0), indicating stronger functional associations between miRNA target genes and immunologic signatures in JIA and CF.

**Figure 6:**
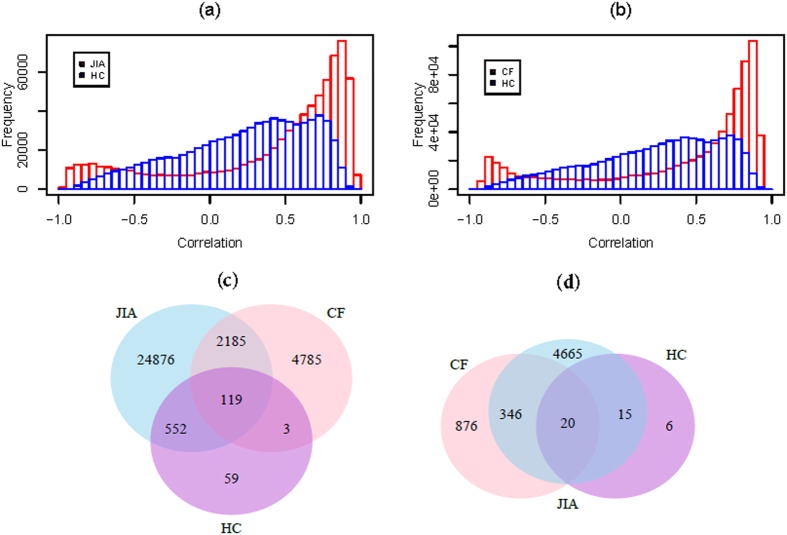
RE-score correlation between miRNA target genes and genes of immunologic signatures from MsigDB. (**a**) Distribution and frequency of Pearson correlation for JIA and HC; (**b**) Distribution and frequency of Pearson correlation for CF and HC. The correlation coefficients from both JIA and CF are significantly larger (*p* = 0) for positive correlation or smaller (*p* = 0) for negative correlation than those from HC; (**c**) Venn diagram showing the number of shared and unique pairs of miRNAs and immunologic signatures with significant positive RE-score correlation (> = 0.9). JIA-CF and JIA-HC display the highest overlap (*p* = 0), followed by CF-HC (*p* = 5.7e-112); (**d**) Venn diagram showing the number of shared and unique pairs of miRNAs and immunologic signatures with significant negative RE-score correlation (< = −0.9). JIA-CF displays the highest overlap (*p* = 0), followed by JIA-HC (*p* = 1.0e-73), and CF-HC (*p* = 3.8e-48).

To obtain significantly correlated miRNAs and immunologic signatures, permutation tests were performed to estimate the *p*-values. Although correlation coefficients of > = 0.7 or < = −0.65 were detected to be significant (*p* < 10^−3^), we established the cutoff to be either > = 0.9 or < = −0.9, since the number of total correlations was large. Using this cutoff, we obtained 27,732, 7092, and 733 positive correlations as well as 5,046, 1242, and 41 negative correlations for JIA, CF, and HC, respectively, substantially more from JIA when compared to CF and HC. The number of shared and unique correlations between JIA, CF, and HC is shown in Fig. 6c,d, where significant overlaps between the three phenotypes were detected from both positive and negative correlations.

A closer look at individual miRNAs in JIA indicates that 4 miRNA clusters of miR-15/16, miR-320, miR-384, and miR-223 are at the top 20 miRNAs that are associated with the largest number (between 248 and 273) of immunologic signatures from positive correlations (Table S5 in Supplementary information). From negative correlations, 7 miRNA clusters, including miR-320 and miR-185, are at the top 20 miRNA list that are associated with 40 to 239 immunologic signatures (Table S5 in Supplementary information). Examining individual immunologic signatures, the top 20 of them are associated with 141 to 173 miRNAs and 78 to 164 miRNAs from positive and negative correlations, respectively (Table S6 in Supplementary information). These immunologic signatures are mainly related to adaptive response immunity^30^,^31^,^32^,^33^,^34^, response to pathogens^35^,^36^, and T cell differentiation^37^.

Taken together, these results indicate that known immunologic signatures have the highest degree of association with miRNA target genes in the JIA neutrophil transcriptome compared with those from CF and HC, suggesting that miRNAs might play important roles in regulating innate immunity in the pathogenesis of JIA^10^,^12^,^13^. As with the miRNA-mRNA correlation analysis, these findings demonstrate again the functional redundancy between JIA and CF phenotypes, exemplifying the idea of common molecular mechanisms mediating gene expression by miRNAs in chronic inflammatory states.

### Validation of gene expression results

**Figure 7:**
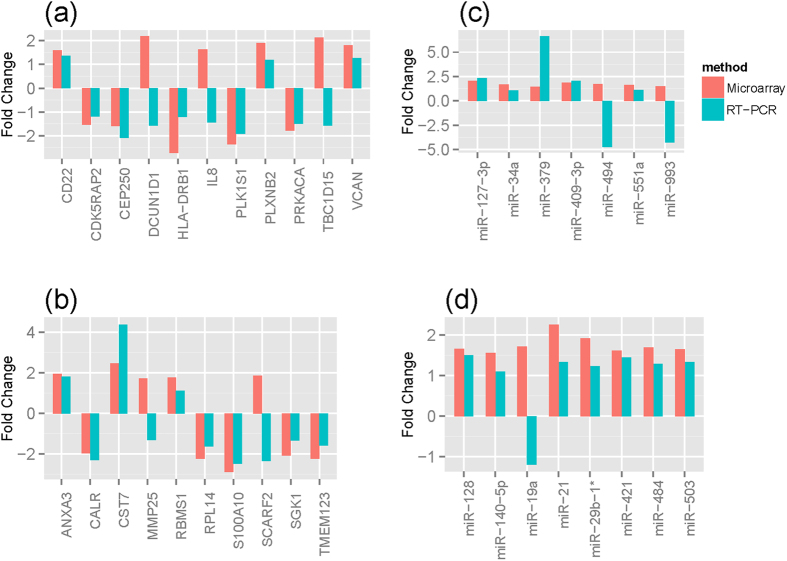
Validation of microarray data for selected genes by real-time PCR. (**a**) Differentially expressed genes from JIA patients were compared to healthy control; (**b**) Differentially expressed genes from cystic fibrosis patients were compared to healthy control; (**c**) Differentially expressed miRNAs from JIA patients were compared to healthy control; (**d**) Differentially expressed miRNAs from cystic fibrosis patients were compared to healthy control.

**Figure 8:**
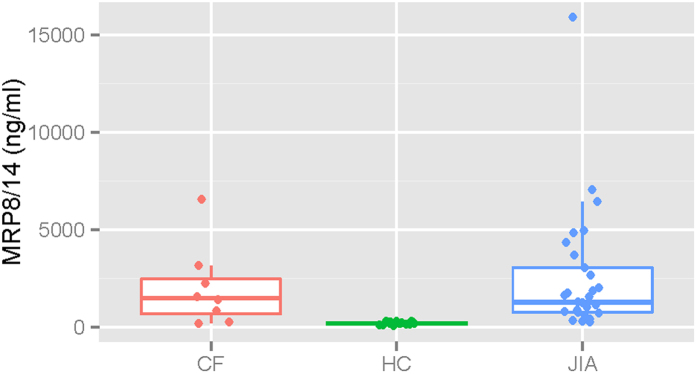
ELISA assays for myeloid-related proteins (MRP) 8/14. – Box plot showing results from ELISA assays for MRP 8/14 in plasma of children with JIA, CF, and healthy controls (HC). Median values for MRP 8/14 were significantly higher (p < 0.001) for CF patients (1,541 ng/ml) and JIA patients (1,473 ng/ml) than for HC (174 ng/ml).

To validate the differences in gene expression observed in the microarray experiments between JIA patients (n = 8) or CF patients (n = 8) and healthy controls (n = 8), we performed RT-PCR analyses by randomly selecting genes and miRNA transcripts that were significantly and differentially expressed. The results indicated that 8 of 11 genes from JIA (Fig. 7a) and 8 of 10 genes from CF (Fig. 7c) had similar expression changes between microarray and RT-PCR. An independent cohort of 4 JIA patients, 4 CF patients and 4 healthy controls were used to validate the differentially expressed miRNAs observed in the microarray experiments by RT-PCR. The results indicated that 5 of 7 miRNAs from JIA (Fig. 7b) and 7 of 8 miRNAs from CF (Fig. 7d) had similar expression changes between microarray and RT-PCR.

### Evidence for chronic neutrophils activation: a comparison of JIA and CF

We have previously presented evidence that circulating neutrophils in children with JIA are in a chronically activated state that is associated with fundamental metabolic properties of the cells^19^. This evidence included aberrant patterns of neutrophil gene expression and elevated levels of myeloid-derived S100 proteins in patient sera. To compare the activation states of JIA and CF neutrophils, we performed ELISA assays for S100 A8/A9 complexes (also known as myeloid-related proteins [MRP] 8/14), potent pro-inflammatory proteins that are sensitive indicators of neutrophil activation^38^ in plasma of children with untreated JIA (n = 31), children with CF (n = 8), and healthy controls (n = 20). JIA and CF patients showed similar MRP8/14 values. The median values for MRP 8/14 complexes were significantly higher (p < 0.001) for CF patients (1,541 ng/ml) and JIA patients (1,473 ng/ml) than for healthy controls (174 ng/ml), as shown in Fig. 8.

## Discussion

One of the most interesting findings from NIH projects like ENCODE and Roadmap Epigenomics has been the elucidation of the complexity eukaryotic transcriptomes. Multiple transcription start sites, gene isoforms, and large and small non-coding RNA transcripts demonstrate that transcription and transcriptional regulation are more complex than would have been anticipated from earlier studies focused on single or small groups of genes. Furthermore, both experimental approaches^26^,^39^ and advanced computational methods have demonstrated that transcriptomes form complex, interactive networks, and that phenotypes^40^, including disease states^41^ and therapeutic response, can be characterized by examining the structure and “rewiring” of these networks^42^,^43^.

In this study, we examined transcriptional complexity in neutrophils of children with 3 distinct phenotypes: juvenile idiopathic arthritis, cystic fibrosis, and healthy children seen in a well-child clinic. We have previously shown that neutrophils of children with JIA show distinct transcriptional abnormalities that are associated with evidence of chronic activation and perturbation of fundamental metabolic processes. These transcriptional abnormalities do not correct when children achieve clinically inactive disease^19^ and maintain that state for 6 consecutive months^13^. Furthermore, contingency analyses of gene array data^19^ suggest that JIA neutrophils have a defect in transcriptional regulation, specifically, those mechanisms associated with coupling and coordination of transcription on a genome-wide basis^44^. Whether these transcriptional aberrations are pathologic, or simply a physiologic adaptation to chronic inflammation in soft tissues was unanswered in our earlier work.

In the current study, we demonstrate that the transcriptional differences seen between neutrophils of children with JIA and healthy children exhibit a degree of specificity while at the same time showing overlap with children with CF, another disease characterized by chronic, indolent inflammation in the soft tissues. At the gene level, we found 216 genes that showed differential expression when we compared children with JIA to HC. However, 148 of these genes were also differentially expressed in children with CF compared with HC, suggesting that the bulk of the transcriptional alterations seen in JIA neutrophils are the predictable result of chronic, soft tissue inflammation. This interpretation is supported by the isoform data; 258 of 296 gene isoforms that were seen in neutrophils of children with JIA, but not in healthy controls, were also detected in children with CF.

A defect in much of the research into JIA and other illnesses associated with chronic, indolent inflammation has been the failure to discern whether abnormalities detected in patients are truly pathological or merely predictable, physiologic adaptations to chronic inflammation in soft tissues. It is easy to forget that, prior to the antibiotic era, chronic inflammation in soft tissues was rather common in humans. It is reasonable to assume that, over time, the immune system has developed mechanisms to keep inflammation localized and limit its systemic effects. Children with CF therefore are informative controls for studies of chronic inflammatory diseases, as we show here.

Despite the considerable degree of overlap between JIA and CF transcriptomes, neutrophils from both CF and JIA patients showed some degree of specificity in genes, gene isoforms, and miRNA expressed. This finding suggests that, while neutrophil responses are typically considered “non-specific,” these cells are capable of making subtle alterations to their transcriptomes to adjust to specific physiologic contexts. To a considerable degree, these alterations are detectable as alternative splicing events. Children with CF, for example, expressed >4,400 isoforms not detected in healthy controls. Thus, while alternative splicing has been shown as a means through which specificity of response is mediated within the adaptive immune system^45^, our data suggest that RNA splicing of this sort is extensive in human neutrophils in the setting of chronic inflammatory states like JIA or CF.

We also demonstrate that the transcriptomes of neutrophils in chronic inflammatory states show extensive network “rewiring”^42^ that is orchestrated by miRNA. The complexities of such miRNA-mRNA networks have been described previously, and even small variations in the number of miRNA molecules or their target mRNAs can have significant effects on network structure^26^,^39^ and protein translation^46^. In this study, we found multiple miRNAs in complex regulatory networks from both JIA and CF. Consistent with the findings from transcriptome analysis, the two phenotypes shared a number of miRNAs and genes in their networks as well as annotated functions. On the other hand, hub miRNA networks were generally distinct to each of the two phenotypes. This finding suggests that neutrophils possess considerable potential to refine physiologic responses in the setting of distinct or even subtly different physiologic milieus. Furthermore, since neutrophils regulate adaptive immunity on multiple levels^14^,^15^, it seems likely that these differences in transcript expression are important elements in determining the ways that neutrophils shape the adaptive immune response.

We are aware of one other paper that uses a network approach to elucidate JIA pathogenesis. Stevens *et al*. used publicly available gene expression and SNP data to construct protein-protein interaction networks for JIA^47^. Such networks are distinctly different from the gene regulatory/co-expression networks we constructed here, as genes can have significant interactions in such networks (e.g., the protein product of gene “A” regulating the gene “B”) without their protein products having significant physical interaction. We note, as well, that the networks constructed by Stevens *et al*. were based, in part, on the assumption that JIA risk-associated SNPs must somehow affect the function of the nearest gene. This isn’t necessarily true. Disease-associated SNPs, even intronic SNPs, may be sites of enhancers that regulate genes many kilobases away^1^,^48^. Even with limitations in comparing the data sets, we note that the Stevens data suggest a role for alternative splicing in JIA pathogenesis, a finding corroborated by our identification of extensive use of splice variants in JIA neutrophils.

These data are limited in that they only provide a static view of what is likely to be a highly dynamic and complex process. Thus, while we have observed surprising complexity in gene interaction networks in neutrophils, and show disease-specific network structures, we lack a definitive understanding of how such networks evolve or emerge over time. It may be impossible to accomplish this aim for juvenile arthritis, an illness where children only present for treatment at the time the disease process is already established. However, by observing transcriptome and network structure and how they are altered by therapy, we are likely to gain valuable insight into mechanisms of therapeutic response, even in settings where therapy does not result in re-establishment of normal immune homeostasis. Furthermore, by observing genes, non-coding transcripts, and regulatory network evolution over the course of therapy, it seems likely that new targets of therapy will be identified. Our research group is currently analysing this kind of dynamic data set generated from children with juvenile arthritis.

In conclusion, we demonstrate here that neutrophils exhibit considerable specificity in their transcriptional repertoire in different pathologic/physiologic contexts. Specificity is seen in different levels, including genes, non-coding miRNAs, isoforms and in the structure of mRNA-miRNA networks where distinct miRNA hubs were observed for each of the disease phenotypes studied here. These findings have interesting implications for our understanding of the mechanisms through which neutrophils shape other elements of immune/inflammatory responses. Furthermore, our findings suggest that, by observing the dynamics of transcriptional repertoire and regulatory network over the course of therapeutic response, we may gain valuable insights into mechanisms of therapeutic response in chronic inflammatory illnesses such as JIA.

## Material and Methods

### Patients and patient characteristics

Patients and controls were recruited from the OU Children’s Physicians’ clinics at the University of Oklahoma Health Sciences Center in Oklahoma City, OK. IRB approval was obtained for this study, which was carried out according to approved guidelines established by the University of Oklahoma Health Sciences Center IRB. Informed consent was obtained from the parents of all patients and controls prior to obtaining specimens; where appropriate, child assent was also obtained.

We studied 35 patients with JIA who had the polyarticular, RF negative phenotype as defined by International League of Associations for Rheumatology (ILAR)^16^. All children had active, untreated disease and were seen within 6 weeks of the onset of symptoms. Findings from children with JIA were compared with findings from 43 healthy control children recruited from the OU Children’s Physician General Pediatrics clinic. In addition to the healthy control children, we studied 15 patients with cystic fibrosis. The cystic fibrosis group represents an important control since it includes children with chronic, usually indolent inflammation of on non-autoimmune origin. This control group allows us to determine those features of the neutrophil transcriptome that are generic to chronic inflammation in soft tissues versus those that are specific to JIA.

## Cell Isolation

Whole blood was drawn into 10 mL citrated CPT tubes (Becton Dickinson, Franklin Lakes, NJ). Specimens were taken immediately to the Pediatric Rheumatology Research laboratories at the University of Oklahoma Health Sciences Center, and cell separation procedures were started within one hour from the time the specimen was drawn. Granulocytes, which sediment with red blood cells in the CPT tubes, were collected and placed in TRIzol reagent immediately (Invitrogen, Carlsbad, CA) after hypotonic lysis of the red blood cells. Cell lysates were stored at −80 °C. Plasma from patients and healthy controls was stored at −80 °C until used in enzyme-linked immunosorbent assays (ELISAs) of MRP8/14 complexes. Cells prepared in this fashion are more than 98% CD66b+  by flow cytometry and contain no contaminating CD14+ cells, as previously reported^19^. Thus, although these cell preparations contained 1–2% CD66b-negative cells, they will be referred to here as “neutrophils” for brevity and convenience.

### RNA isolation, labeling, and gene expression profiling

Total RNA was extracted from Trizol® reagent according to the manufacturer’s directions, including a DNAse (Qiagen) step to remove genomic DNA. RNA was quantified spectrophotometrically (Nanodrop) and assessed for quality by capillary gel electrophoresis (Agilent 2100 Bioanalyzer; Agilent Technologies, Inc., Palo Alto, CA) to determine the ratio of 28s:18s rRNA in each sample. A ratio greater than 1.0 was used to define samples of sufficient quality, and only samples above this limit were used for microarray studies.

RNA samples were processed using GeneChip WT Terminal Labeling and Controls Kit and hybridized to Human Exon 1.0 ST array according to the manufacturer’s protocol (Affymetrix, Santa Clara, CA, USA). The GeneChip™ miRNA array (Affymetrix®, Santa Clara, CA) platform was used to quantify miRNAs in total RNA. RNA samples were processed using FlashTag Biotin HSR kit and hybridized to human GeneChip microRNA Galaxy Array (Affymetrix, Santa Clara, CA, USA). GeneChips™ were washed and stained using an Affymetrix automated GeneChip™ 450 fluidics station and scanned with an Affymetrix 3000 7G scanner.

### GeneChip data processing and analysis

To generate expression summary values from Affymetrix Exon and miRNA arrays, *RMA* software in the “Affy” package of Bioconductor in the *R* statistical computing environment (http://www.r-project.org) was used with its default settings. For exon array the library of exon.pmcdf was used and the resulting expression values from RefSeq genes were selected for further analysis. Differentially expressed genes (*p* < = 0.001 and fold change > = 1.5) and miRNAs (FDR < = 0.1) between different phenotypes were obtained using *t*-tests. For multiple test correction, the false discovery rate (miRNA: *q*-value < = 0.1) was controlled by adjusting the *p*-values using the following formula:


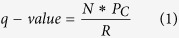


where *N* is the number of genes/miRNAs in the test, and *R* is the ascending rank order of the respective *p*-value at certain cutoff *P*_*c*_.

To identify isoforms displaying differential exon splicing, Affymetrix power tools (www.affymetrix.com) and R statistical software were employed to compare splicing differences between phenotypes as described by Lockstone^49^. Both *p*-values (*p* < = 0.05) from the program MiDAS and Splicing Index (SI > = 1.2) were used to determine isoforms displaying differential exon splicing. The latter was computed using an in-house developed PERL script based on the definition of the Splicing Index.

### Unsupervised hierarchical clustering

To visualize the expression pattern of differentially expressed genes across JIA and HC samples, unsupervised hierarchical clustering analysis was carried out using heatmap.2 function in R package. Prior to the hierarchical clustering, expression values of individual genes across all JIA and HC samples were first normalized to the median value. Hierarchical clustering of the expression profile was then done using average linkage and Euclidean distances.

### Correlation analysis between mRNA and miRNA expression profiles

To compute the correlation between mRNA expression profiles and miRNA expression profiles, genes in the mRNA expression profiles were first mapped to the target genes of individual miRNAs by TargetScan^22^, which lists 239 miRNAs and 12200 unique target genes. The expression values for each of these 239 miRNAs from miRNA expression profiles were then paired to the mRNA expression of its target genes in matched samples, and the resulting miRNA-mRNA expression pairs were used for computing correlation (Both Pearson and Spearman correlation coefficients). To estimate the statistical significance of a correlation, permutation tests were first performed to create 1,000 datasets by randomizing samples of miRNA or mRNA expressions from genes of the test datasets. Each of the random datasets was subjected to correlation analysis, and the resulting correlations were then compared to the correlation from test data set. The number of times that correlation coefficients from random data sets is smaller than actual correlation coefficient over N = 1000 was taken as the *p*-value. All analyses were performed using in-house developed PERL scripts.

### Correlation between miRNA target genes and immunologic gene signatures

To investigate the functional association of miRNAs with immunologic signatures, we downloaded 1910 gene sets of immunologic signatures from Broad Institute (http://www.broadinstitute.org). These gene sets, which represent cell states and perturbations within the immune system, were defined directly from microarray gene expression data from immunologic studies. We then employed a regulatory effect score (RE-score)^29^, which is based on rank comparison, to measure the difference in expression levels between genes of individual immunologic signature and gene of non-immunologic signature. This was done by first rank ordering the gene expression values in individual samples, followed by computing the average rank of genes for individual immunologic signature and the average rank of the rest of the genes in the transcriptome. The RE-score was calculated as the difference between the 2 average ranks. The same method was applied to compute RE-scores for individual miRNAs, except that miRNA target genes were used. The resulting RE-scores from each of the 239 miRNAs were mapped to the RE-scores from each of the 1910 immunologic signatures in matched samples. Both Pearson and Spearman correlation coefficients were then computed. To estimate the statistical significance of a correlation (*p* < = 0.001), permutation tests were performed to create 1,000 datasets by randomizing the order of miRNA target gene or the signature genes in the test gene expression dataset. Each of the random datasets was subjected to correlation analysis, and the resulting correlations were then compared to the correlation from real RE-scores. The number of times that correlation coefficients from random data sets is bigger (for positive correlations) or smaller (for negative correlations) than actual correlation coefficient over N = 1000 was taken as the *p*-value. In-house developed PERL scripts and R packages were used for the analyses.

### Gene expression validation by quantitative real-time RT-PCR

For validation purposes we analysed the expression of single mRNAs and miRNA transcripts using quantitative real time-polymerase chain reaction (RT-PCR) in the same samples as used for microarray. Total RNA was reverse transcribed with iScript™ cDNA synthesis kit according to the directions of the manufacturer (Bio-Rad, Hercules, CA, USA). Real-time RT-PCR was performed using SYBR Green reagents on a StepOne Plus (for the testing group; Applied Biosystems, Foster City, CA, USA) as described previously^13^. Gene amplification was performed using specific primers (Table S7 in Supplementary information). Gene-specific amplification was confirmed by a single peak in the ABI Dissociation Curve software. Average threshold cycle (Ct) values for GAPDH (run in parallel reactions to the genes of interest) were used to normalize average Ct values of the gene of interest. These values were used to calculate averages for each group (healthy control or patient subsets), and the relative ΔCt was used to calculate fold-change values between groups. All primers applied were tested to display an efficiency of amplification approximate 98% (±SD 4.65%).

To validate miRNA expression, quantitative real time RT-PCR was performed using the TaqMan MicroRNA Reverse transcription kit (Applied Biosystems, USA), miRNA-specific stem-loop primers (TaqMan® microRNA assay kit, Applied Biosystems, USA) and the TaqMan Universal Master Mix II, no UNG (Applied Biosystems, USA), as per the manufacturers’ instructions. The 20-μl reaction mix required for the quantitative PCR contained 1.2 μl of the product from the RT reaction, 10 μl of TaqMan Universal Master Mix II, no UNG, 1 μl of TaqMan miRNA assay (20x), and 7.8 μl nuclease-free water. PCRs for each individual miRNA were carried out in duplicate using the StepOne Plus PCR system (Applied Biosystems) with specific target sequence (Table S8 in Supplementary information). The temperature profile consisted of an initial step at 95 °C for 10 minutes, followed by 40 cycles of 95 °C for 15 seconds, 60 °C for 1 minute. 2 miRNAs of hsa-miR-191 and hsa-miR-16 were identified as miRNA normalizers, since they displayed no statistically significant difference among groups, had the smallest variation across all 86 samples (CV = 1.2 and 1.8%), and had relatively high expression values. Average threshold cycle (Ct) values from the 2 miRNAs (running in parallel reactions to the miRNA of interest) were used to normalize average Ct values of the miRNAs of interest. These values were used to calculate averages for each group (healthy control or patient subsets), and the relative ΔCt was used to calculate fold-change values between the groups.

### Determination of MRP8/14 plasma level

Plasma levels of MRP8/14 complexes were measured by sandwich ELISA (Bühlmann Laboratories, Schönenbuch, Zwitserland) following the manufacturer’s instructions. In brief, plasma samples were diluted with incubation buffer (patient plasma, 1:100; healthy control plasma, 1:1). One hundred microliters of each diluted sample and 100 μl high and low serum control (provided with the kit) was added into the well of the coated, prewashed plate. The well was washed three times after incubation for 30 minutes at room temperature. Wells were then incubated with anti-MRP8/14 Ab conjugated to HRP for 30 minutes followed by washing. One hundred micro liters of TBA substrate was added to eachwell and incubated for 15 minutes. The absorbance at 450nm was read using a microplate reader.

## Additional Information

**How to cite this article**: Hu, Z. *et al*. Complexity and Specificity of the Neutrophil Transcriptomes in Juvenile Idiopathic Arthritis. *Sci. Rep*. **6**, 27453; doi: 10.1038/srep27453 (2016).

## Supplementary Material

Supplementary Information

## Figures and Tables

**Table 1:** Differentially expressed miRNA transcripts.

miRNA	Mean JIA (log2)	Mean HC (log2)	*p*-value	Fold Change	FDR	Unique to JIA or common between JIA and CF
miR-34a	3.005	2.273	6.47E-06	1.660940048	5.48E-03	unique
miR-936	1.34	1.628	1.44E-04	−1.220946513	2.44E-02	unique
miR-127-3p	3.014	1.995	9.03E-05	2.0265138	2.55E-02	unique
miR-409-3p	3.766	2.871	1.28E-04	1.859609885	2.70E-02	unique
miR-933	3.147	2.57	1.93E-04	1.491744027	2.73E-02	unique
miR-379	2.106	1.566	2.27E-04	1.453972517	2.75E-02	unique
miR-494	4.193	3.396	2.60E-04	1.73748437	2.75E-02	common
miR-551a	3.438	2.713	7.40E-05	1.652900636	3.13E-02	common
miR-1285	3.998	3.412	9.92E-04	1.501079098	9.34E-02	unique
