# Supplementary material for: Complexity and Specificity of the Neutrophil Transcriptomes in Juvenile Idiopathic Arthritis
Source: Sci Rep. 2016 Jun 7;6:27453. doi: 10.1038/srep27453 (PMC4895221; doi:10.1038/srep27453)
Supplement: Supplementary Information [file srep27453-s1.pdf]

Supplementary Figures and Tables

Manuscript number: SREP-15-19125
Title*: Complexity and Specificity of the Neutrophil Transcriptomes in Juvenile Idiopathic Arthritis*

Zihua Hu, Kaiyu Jiang, Mark Barton Frank, Yanmin Chen, James N. Jarvis

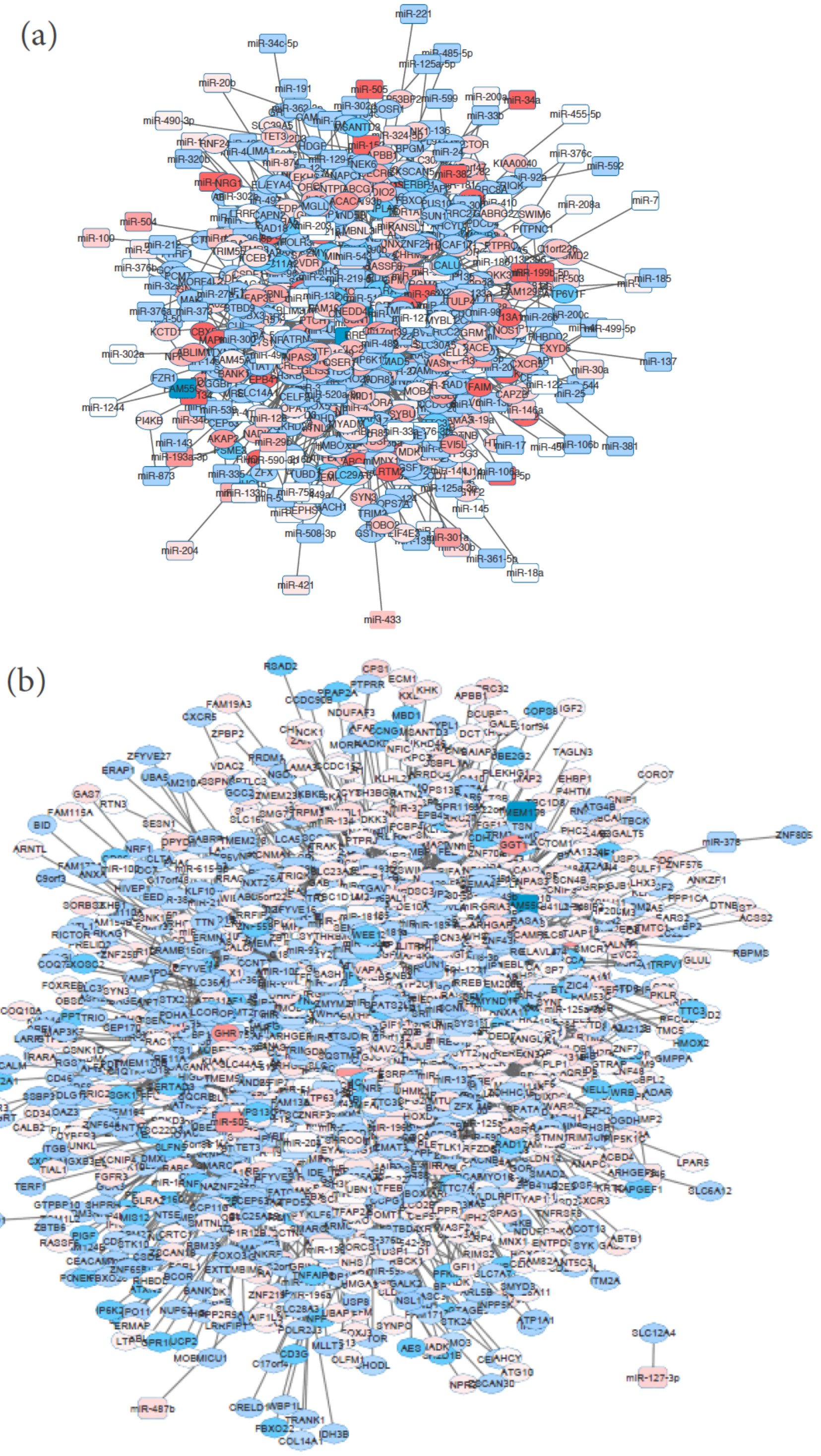

Figure S1. Overall integrated miRNA-gene regulatory network. Graphical representation of regulatory network for JIA (a) and CF (b). Rectangles: miRNAs. Circles: target genes. Red: up-regulated miRNAs and target genes when compared to HC. Blue: down-regulated miRNAs and target genes when compared to HC.

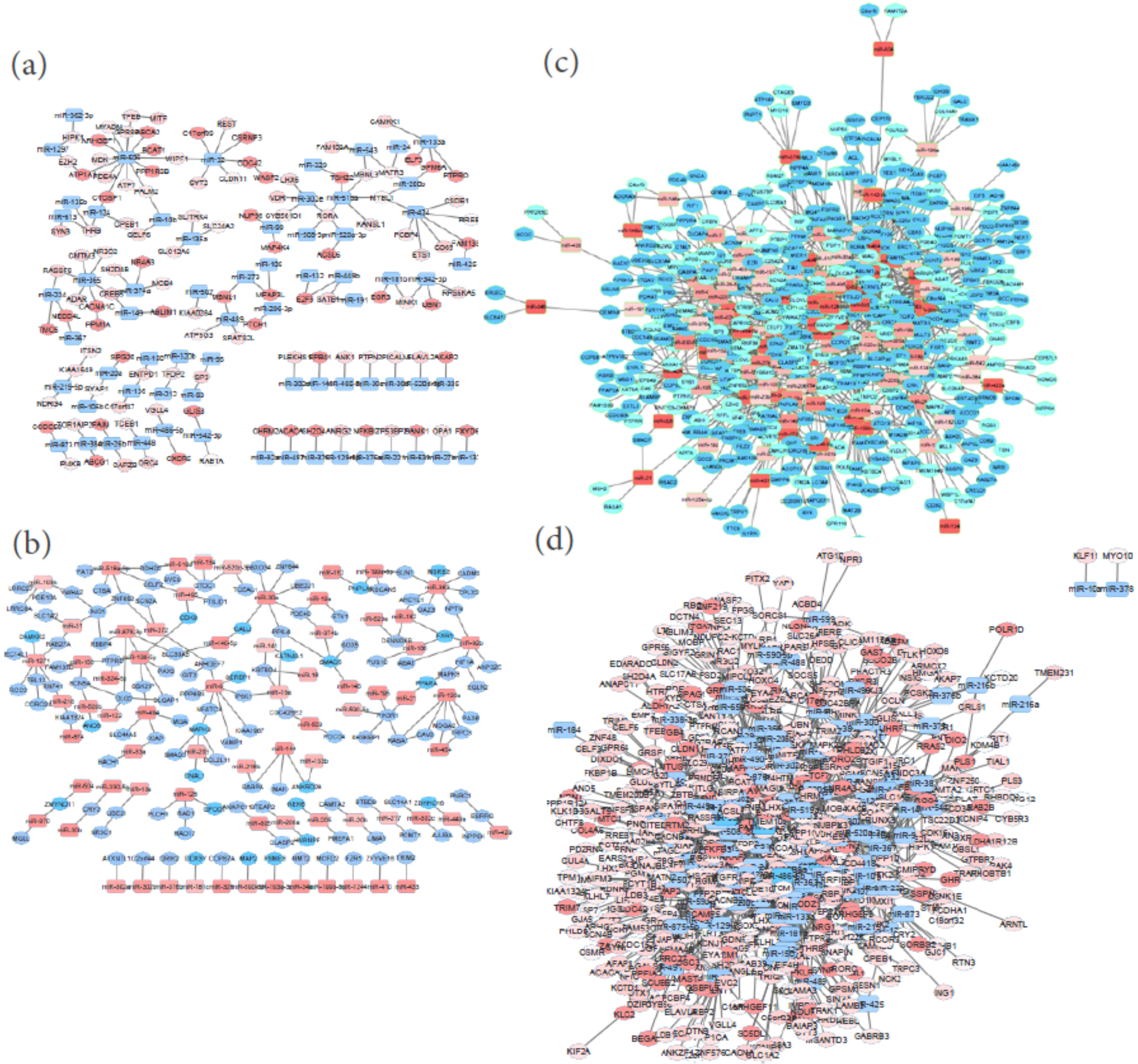

Figure S2. Integrated miRNA-gene regulatory network with the expression of miRNAs and their target genes in the opposite directions. Graphical representation of regulatory networks from up-regulated miRNAs and down-regulated target genes in JIA (a) and CF (c). Graphical representation of regulatory networks from down-regulated miRNAs and up-regulated target genes in JIA (b) and CF (d). Rectangles: miRNAs. Circles: target genes. Red: up-regulated miRNAs and target genes when compared to HC. Blue: down-regulated miRNAs and target genes when compared to HC.

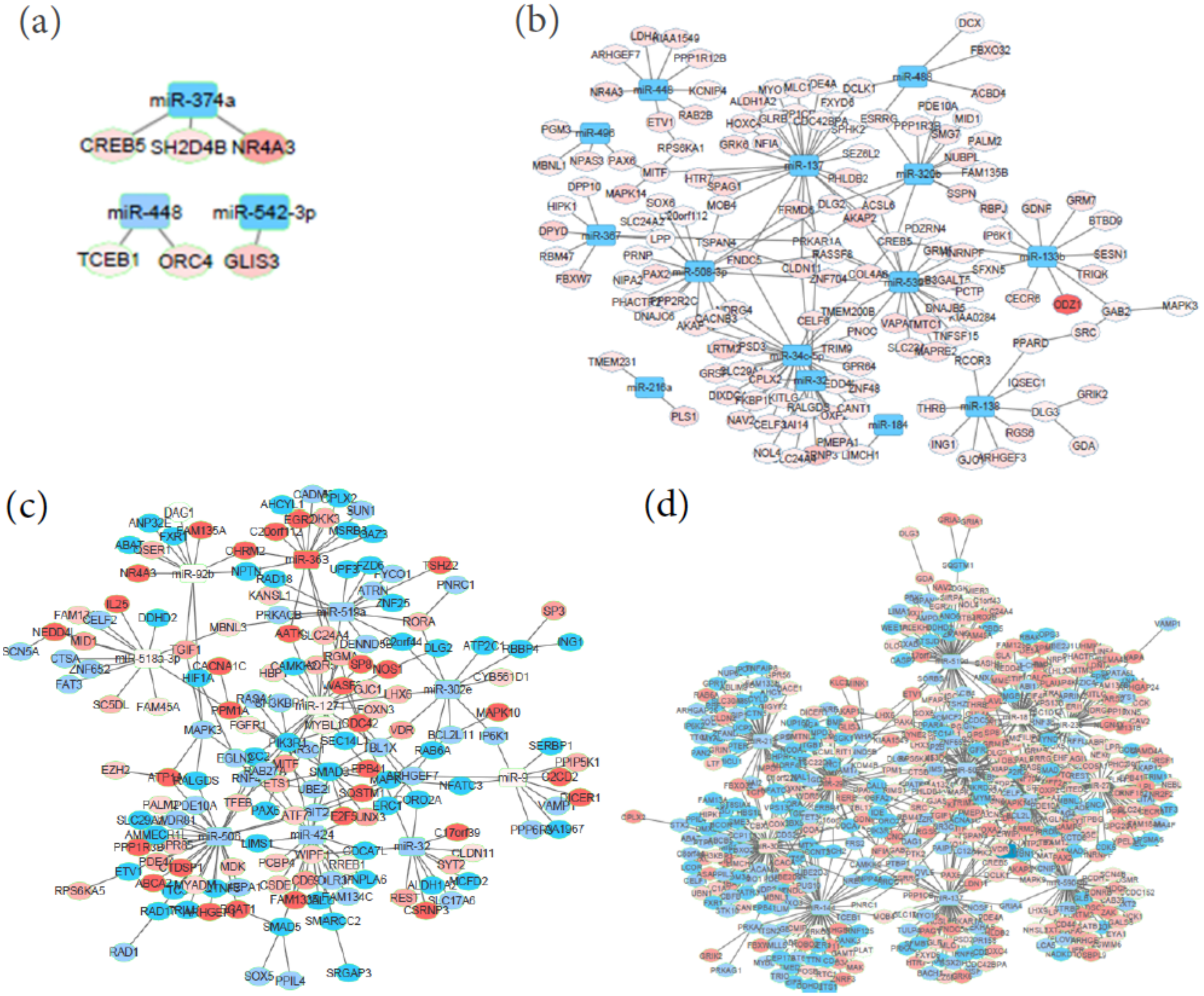

Figure S3. Integrated miRNA-gene regulatory networks from hub miRNAs. Graphical representation of regulatory network in JIA (a) and CF (b). Rectangles: miRNAs. Circles: target genes. Red: up-regulated miRNAs and target genes when compared to HC. Blue: down-regulated miRNAs and target genes when compared to HC.

Table S1: 216 differentially expressed genes between JIA and HC

Gene Symbol	Mean (JIA)	Median (JIA)	stdev (JIA)	Mean (HC)	Median (HC)	stdev (HC)
ACER3	5.014	5.019	0.511	5.625	5.621	0.78
ACPL2	3.669	3.4	0.911	4.405	4.292	0.866
AP2M1	10.223	10.211	0.384	9.401	9.249	0.752
APBB2	4.14	4.112	0.742	3.553	3.615	0.756
ASNSD1	7.003	7.038	0.477	6.369	6.606	1.026
BRWD3	7.903	7.817	0.62	7.22	7.175	0.613
C21orf33	5.55	5.607	0.509	4.877	4.81	0.611
CD22	5.116	5.167	0.702	4.452	4.675	0.833
CD9	8.267	8.243	0.643	8.982	8.885	0.967
CDK14	5.494	5.608	0.677	4.872	4.965	0.751
CHRFAM7A	7.304	7.339	0.529	7.913	7.909	0.825
CREB5	7.912	7.923	0.65	7.29	7.328	0.609
CYP26B1	5.581	5.461	0.72	4.902	4.851	0.653
DCUN1D1	8.672	8.845	0.742	7.544	7.772	0.994
DERA	2.394	2.255	0.791	3.052	3.017	0.905
ELF5	7.249	7.333	1.268	8.164	8.018	1.008
ENTPD7	7.042	7.065	0.82	7.639	7.676	0.451
EPB41	4.678	4.689	0.788	3.945	4.043	0.899
H2AFY	4.723	4.56	0.689	4.133	4.106	0.678
HLA-DRB1	3.708	3.476	1.152	5.151	4.699	1.697
HMGN3	6.827	6.808	0.969	5.651	5.728	1.055
IDNK	4.997	5.073	0.871	5.712	5.768	0.684
IL8	11.904	12	0.682	11.195	11.475	0.862
IQGAP2	7.454	7.405	0.643	6.817	6.696	0.835
ITGB1BP2	4.461	4.439	0.704	5.062	5.058	0.489
KHDRBS2	4.854	4.639	0.98	4.075	3.933	1.003
KIAA0930	7.446	7.683	0.748	8.038	8.141	0.494
LHCGR	2.387	2.134	1	3.291	3.177	1.226
LRP1	7.206	7.222	0.7	6.585	6.697	0.715
LRRC29	3.251	3.237	0.72	3.868	3.822	0.632
MAN1A2	8.532	8.565	0.613	7.828	7.821	0.411
MAPKAPK2	5.905	5.843	0.478	5.283	5.35	0.583
MED27	6.379	6.513	0.784	6.966	6.982	0.469
MLL3	11.102	11.192	0.291	10.502	10.413	0.312
MLLT4	6.245	6.263	1.038	5.403	5.552	1.053
NFAT5	3.715	3.727	0.643	4.311	4.436	0.844
PCDH8	3.415	3.477	0.751	4.451	4.308	1.379
PCNA	2.619	2.477	0.764	3.214	3.203	0.726
PLXNA2	6.864	6.835	0.409	6.205	6.411	0.912
PLXNB2	5.612	5.72	0.867	4.689	4.935	1.244
PRKACA	8.336	8.186	0.706	9.165	9.418	0.763
PRKAR2B	7.718	7.501	1.119	6.552	6.543	0.934
PSG4	3.499	3.491	0.972	4.381	4.594	1.07
PSG8	4.62	4.763	0.748	5.214	5.318	0.737

RHOT2	4.615	4.609	0.64	5.235	5.383	0.709
ROMO1	8.515	8.615	0.466	7.721	7.731	0.723
RPL10	11.716	11.784	0.56	10.964	11.38	1.151
SLC25A19	3.712	3.939	0.9	2.941	2.785	0.855
SMARCAL1	4.202	4.271	0.846	4.836	4.9	0.698
SNX18	6.263	6.294	0.769	6.852	6.804	0.481
SSNA1	6.562	6.549	0.583	7.163	7.204	0.37
STIL	3.731	3.899	0.901	3.088	2.986	0.678
SUSD4	4.359	4.538	0.909	5.038	4.992	0.53
TBC1D15	3.857	3.977	1.224	2.767	2.399	1.457
TENC1	3.453	3.464	0.394	4.05	4.013	0.527
TGM3	7.042	7.088	0.711	6.393	6.413	0.821
THNSL2	2.874	2.877	0.519	3.673	3.494	0.902
TPGS2	2.257	1.925	1.125	3.292	3.36	1.382
TPM3	4.493	4.547	0.79	3.75	3.72	0.903
TRIM37	4.034	4.133	0.891	4.728	4.78	0.661
UBE2M	4.465	4.639	0.858	5.153	5.131	0.564
VCAN	6.507	6.413	0.851	5.651	5.804	1.08
VIP	2.525	2.56	0.618	3.11	3.092	0.743
WDR20	7.772	7.855	0.407	6.947	6.843	0.558
XRCC5	5.699	5.855	0.749	5.096	5.249	0.694
ZMAT2	7.792	7.827	0.294	7.194	7.143	0.42
ZNF107	9.499	9.439	0.888	8.739	8.593	0.813
ZNF180	4.573	4.656	0.753	5.317	5.239	0.55
AAMP	5.618	5.517	0.435	6.236	6.322	0.525
ABCA13	7.949	7.986	0.433	7.296	7.431	0.645
ABCB10	4.686	5.171	1.262	5.616	5.683	0.957
ACSF2	6.009	6.011	0.755	6.644	6.57	0.827
AFF1	5.658	5.682	0.765	4.745	5.101	1.31
AGGF1	3.671	3.507	0.909	4.403	4.538	0.761
ALDH6A1	2.765	2.713	0.835	3.62	3.59	1.23
ANAPC4	3.744	3.658	0.77	4.445	4.358	0.802
ANXA1	7.622	7.631	0.708	8.461	8.537	0.78
ANXA6	6.996	6.807	0.762	7.741	7.833	0.812
AP2A1	6.474	6.565	0.706	7.078	7.138	0.377
APOOL	6.331	6.419	0.681	6.93	6.93	0.434
ARMC10	6.649	6.737	0.621	7.241	7.222	0.636
AS3MT	4.496	4.557	0.786	5.207	5.292	0.719
ASRGL1	5.619	5.673	0.716	6.213	6.174	0.808
ASTE1	4.592	4.65	0.632	5.198	5.18	0.61
ATAD5	2.549	2.503	0.616	3.142	3.223	0.714
ATP6V0A2	4.33	4.42	0.773	4.939	4.989	0.753
ATXN2	5.108	5.347	0.93	5.733	5.751	0.564
BCAS3	8.981	9.059	0.548	9.647	9.519	0.585
BRD7	5.454	5.635	0.948	6.109	6.196	0.625
BTK	7.845	7.944	0.635	8.497	8.477	0.398
C10orf88	4.599	4.45	0.889	5.285	5.388	0.864
CAB39L	4.39	4.438	0.84	4.999	5.104	0.617

CCDC53	5.653	5.715	0.778	6.239	6.265	0.466
CCDC7	4.796	4.767	0.93	5.438	5.418	0.611
CCDC83	5.556	5.583	0.645	4.917	4.979	0.687
CCR3	6.603	6.765	1.161	7.559	7.596	0.8
CCT5	5.711	5.618	0.651	6.298	6.344	0.488
CD164	8.64	8.805	0.797	9.232	9.232	0.496
CD300LB	5.868	5.874	0.771	6.532	6.527	0.664
CD300LF	8.819	8.905	0.614	9.417	9.344	0.54
CD55	7.892	7.723	0.631	7.288	7.455	0.64
CD84	3.751	3.764	0.967	4.548	4.593	0.757
CDC16	5.904	5.812	0.9	6.626	6.66	0.654
CDK5RAP2	5.951	6.043	0.619	6.566	6.445	0.592
CEP250	4.696	4.672	0.846	5.365	5.296	0.756
CLN5	5.544	5.635	0.686	6.149	6.098	0.545
CLTCL1	5.603	5.592	0.651	5.01	5.089	0.829
COG7	5.309	5.356	0.72	5.9	5.871	0.408
CRYZL1	5.21	5.069	0.826	5.937	5.747	0.549
CS	6.465	6.598	0.694	7.061	6.969	0.654
CTNS	4.68	4.769	0.458	5.278	5.224	0.71
CYB5B	4.994	5.058	0.716	5.714	5.764	0.651
DAAM1	4.2	4.203	0.974	4.988	5.092	0.838
DENND1B	7.073	7.045	0.685	7.668	7.831	0.635
DIP2A	6.229	6.298	0.818	6.869	6.889	0.515
DNAL1	1.88	1.7	0.804	2.61	2.505	1.019
DOK2	7.665	7.668	0.654	8.272	8.13	0.505
DRAP1	11.25	11.384	0.57	10.627	10.618	0.565
EBP	4.354	4.398	1.028	5.432	5.445	0.757
ECHDC3	6.35	6.238	0.58	5.752	5.78	0.691
EFTUD1	4.319	4.174	0.816	4.971	4.964	0.808
EMR1	10.249	10.394	0.841	11.208	11.278	0.824
ERP29	6.257	6.364	0.866	6.874	6.921	0.575
FAIM	5.82	5.763	0.531	5.191	5.292	0.776
FAM63B	7.381	7.41	0.515	7.979	8.006	0.41
FAM82B	6.268	6.36	0.759	6.881	6.973	0.519
FNBP4	7.706	7.58	0.596	8.304	8.329	0.418
FUCA1	7.049	6.941	0.647	7.685	7.708	0.507
G2E3	5.713	5.767	0.759	6.421	6.416	0.513
GART	5.559	5.715	0.59	6.204	6.225	0.466
GIN1	4.632	4.765	0.687	5.255	5.329	0.621
GPR19	4.339	4.28	0.723	4.964	5.027	0.861
HAX1	6.906	6.976	0.77	7.539	7.568	0.453
HCK	10.005	10.024	0.233	9.372	9.405	0.392
HMG20B	6.073	6.185	0.71	6.741	6.717	0.477
IFT80	3.832	3.846	0.915	4.684	4.815	0.954
IL3RA	5.623	5.628	0.729	6.318	6.218	1.024
IMPDH1	8.463	8.428	0.416	7.844	7.904	0.498
INO80C	6.377	6.501	0.645	7.017	7.126	0.497
INPP1	5.714	5.842	0.707	6.329	6.292	0.858

INPP5B	8.473	8.371	0.636	9.088	9.096	0.529
INPP5F	2.713	2.645	1.094	3.859	4.073	1.18
KANSL2	5.135	5.28	0.86	5.749	5.749	0.478
KIF13B	5.788	5.815	0.699	6.69	6.804	0.907
LILRA2	10.315	10.389	0.316	9.727	9.638	0.469
LOC100505679	4.916	5.112	0.759	5.608	5.716	0.79
LOC81691	3.576	3.429	0.844	4.292	4.326	0.917
LRIG1	5.173	5.345	0.69	5.782	5.813	0.676
LRRC6	10.496	10.614	0.509	9.859	10.036	0.917
MCM3AP	6.942	7.188	0.825	7.558	7.585	0.412
METTL10	5.158	5.347	1.036	5.908	5.927	0.645
MRPL49	4.856	4.77	0.734	5.565	5.646	0.622
MSANTD3	6.134	6.116	0.608	6.745	6.886	0.597
NBEAL1	3.805	3.491	1.19	4.817	5.177	1.25
NDUFAF1	5.135	5.267	0.876	5.81	5.854	0.53
NTAN1	6.05	5.994	0.776	6.7	6.551	0.629
NUP107	3.761	3.69	0.892	4.486	4.571	0.703
NUP85	6.743	6.808	0.612	7.396	7.4	0.473
ODZ1	6.286	6.517	1.011	5.09	5.197	1.421
PARVB	5.422	5.308	0.693	4.835	4.793	0.685
PCBD2	3.462	3.564	0.659	4.061	4.131	0.544
PFKM	3.887	3.879	0.678	4.735	4.641	0.877
PIBF1	3.71	3.772	0.743	4.31	4.327	0.704
PLK1S1	7.822	7.108	1.464	9.054	9.344	1.107
POMT1	5.077	5.311	1.045	5.873	5.871	0.537
PPP2CB	3.921	3.784	0.955	4.627	4.616	0.721
PPP2R4	5.46	5.514	0.53	6.088	6.2	0.43
PRDM15	4.974	5.095	1.011	5.707	5.814	0.708
PRR5L	6.474	6.487	0.904	7.182	7.312	0.768
PSPH	5.162	5.182	1.16	6.197	6.657	1.209
PTPN1	5.433	5.462	0.661	6.063	6.143	0.564
RAD23B	7.35	7.465	0.424	6.504	6.47	0.865
RBM14	8.57	8.541	0.443	9.198	9.312	0.382
RECQL	5.371	5.64	0.895	6.009	5.956	0.559
REEP4	4.828	5.067	0.79	5.447	5.525	0.704
RNF5	8.315	8.396	0.532	9.014	8.927	0.383
RSPH10B	2.711	2.522	0.593	3.325	3.463	0.527
RYK	5.364	5.578	0.78	5.95	5.978	0.531
S100PBP	5.572	5.648	0.768	6.169	6.298	0.53
S100Z	7.431	7.539	0.53	8.021	7.968	0.525
SCP2	5.641	5.858	0.997	6.454	6.396	0.86
SETDB2	5.836	5.917	0.875	6.426	6.511	0.526
SFI1	6.229	6.206	0.832	6.82	6.876	0.543
SKA2	5.149	5.187	0.71	5.736	5.855	0.582
SLC16A3	10.116	10.054	0.622	9.277	9.307	0.346
SLC19A1	8.361	8.5	0.508	7.687	7.727	0.331
SLC25A25	5.114	5.077	0.562	5.709	5.764	0.675
SLC25A30	4.814	4.802	1.019	5.698	5.766	0.624

SLFN12	6.344	6.1	0.809	7.022	7.127	0.919
SMN1	5.259	5.165	0.577	5.878	5.897	0.872
SMPDL3A	4.131	4.159	0.865	3.45	3.42	0.756
SNRNP25	6.119	6.146	0.8	6.818	6.893	0.925
SPPL2A	7.653	7.869	0.785	8.242	8.244	0.444
STXBP5	7.819	7.807	0.444	8.606	8.682	0.655
SUGP2	4.847	4.961	0.816	5.442	5.481	0.538
TAS2R31	2.539	2.489	0.605	3.145	3.027	0.849
TAX1BP3	7.709	7.618	0.557	8.336	8.419	0.503
TCEB3C	4.014	4.028	0.502	3.403	3.456	0.663
TEC	5.56	5.427	0.845	6.292	6.181	0.983
TMEM216	6.153	6.16	0.591	6.753	6.791	0.474
TMEM50B	7.396	7.423	0.686	7.981	7.914	0.67
TMEM68	5.6	5.71	0.785	6.205	6.169	0.545
TMX1	4.554	4.671	0.946	5.36	5.473	0.6
TRAPPC4	5.397	5.517	0.764	6.07	6	0.444
UCK1	8.19	8.19	0.36	8.87	8.996	0.46
ULK4	4.493	4.58	1.106	5.336	5.393	1.015
UPRT	5.295	5.108	0.713	5.973	6.128	0.631
VPS33A	4.501	4.519	0.781	5.24	5.36	0.63
WDTC1	9.434	9.546	0.312	8.689	8.651	0.351
YTHDC1	9.62	9.614	0.306	8.994	8.847	0.494
ZDHHC13	5.919	5.935	0.648	6.528	6.532	0.427
ZMYND8	6.912	7.059	0.566	7.532	7.595	0.409
ZNF28	3.147	3.085	0.795	3.825	3.796	0.862
ZNF395	6.655	6.709	0.666	7.253	7.187	0.665
ZNF750	4.008	4.166	0.784	3.335	3.411	0.862
ZSCAN29	5.148	5.07	0.944	6.033	6.04	0.799

p-value	Fold Change (JIA vs HC)	FDR	Unique to JIA or common between JIA and CF
8.61E-05	-1.528	0.0133	unique
5.39E-04	-1.665	0.037	unique
3.79E-08	1.768	0.0001	unique
9.47E-04	1.502	0.0502	unique
6.26E-04	1.552	0.0402	unique
6.51E-06	1.605	0.0029	unique
1.05E-06	1.595	0.001	unique
2.74E-04	1.584	0.0254	unique
2.11E-04	-1.641	0.0222	unique
2.54E-04	1.539	0.0245	unique
1.84E-04	-1.525	0.0205	unique
4.87E-05	1.54	0.0096	unique
5.11E-05	1.601	0.0098	unique
1.97E-07	2.184	0.0003	unique
9.87E-04	-1.579	0.0512	unique
9.45E-04	-1.885	0.0501	unique
3.29E-04	-1.512	0.0281	unique
2.55E-04	1.663	0.0245	unique
3.11E-04	1.505	0.0273	unique
2.93E-05	-2.719	0.0072	unique
2.28E-06	2.259	0.0016	unique
1.88E-04	-1.642	0.0208	unique
1.18E-04	1.635	0.0159	unique
2.91E-04	1.554	0.0263	unique
6.91E-05	-1.517	0.0118	unique
9.18E-04	1.716	0.0493	unique
1.72E-04	-1.507	0.0197	unique
5.89E-04	-1.871	0.0389	unique
2.42E-04	1.538	0.0238	unique
1.71E-04	-1.534	0.0197	unique
2.89E-07	1.629	0.0004	unique
1.82E-06	1.538	0.0014	unique
2.76E-04	-1.502	0.0255	unique
4.08E-13	1.516	0	unique
7.00E-04	1.793	0.0428	unique
6.95E-04	-1.511	0.0426	unique
7.56E-05	-2.05	0.0123	unique
8.13E-04	-1.511	0.0464	unique
7.60E-05	1.579	0.0124	unique
2.47E-04	1.897	0.0241	unique
4.11E-06	-1.776	0.0023	unique
5.89E-06	2.245	0.0028	unique
2.83E-04	-1.844	0.0258	unique
7.73E-04	-1.51	0.0453	unique

1.23E-04	-1.537	0.0162	unique
1.25E-07	1.735	0.0003	unique
3.60E-04	1.684	0.0295	unique
2.57E-04	1.706	0.0246	unique
6.90E-04	-1.553	0.0424	unique
2.28E-04	-1.505	0.0231	unique
2.16E-06	-1.517	0.0015	unique
8.86E-04	1.562	0.0485	unique
2.71E-04	-1.6	0.0253	unique
5.84E-04	2.128	0.0387	unique
2.05E-07	-1.512	0.0003	unique
3.55E-04	1.568	0.0293	unique
6.14E-06	-1.74	0.0028	unique
4.83E-04	-2.05	0.0348	unique
2.27E-04	1.674	0.0231	unique
3.02E-04	-1.618	0.0269	unique
1.45E-04	-1.61	0.0178	unique
1.96E-04	1.81	0.0212	unique
2.92E-04	-1.5	0.0263	unique
8.89E-11	1.772	0	unique
4.95E-04	1.519	0.0354	unique
1.79E-10	1.514	0	unique
2.15E-04	1.694	0.0224	unique
7.95E-06	-1.676	0.0033	unique
2.38E-07	-1.534	0.0004	common
1.03E-06	1.573	0.001	common
6.30E-04	-1.906	0.0404	common
6.98E-04	-1.552	0.0427	common
2.70E-04	1.883	0.0252	common
3.15E-04	-1.661	0.0276	common
5.00E-04	-1.809	0.0356	common
1.91E-04	-1.626	0.021	common
4.11E-06	-1.788	0.0023	common
8.08E-05	-1.677	0.0128	common
3.40E-05	-1.52	0.0078	common
3.36E-05	-1.515	0.0078	common
9.00E-05	-1.507	0.0137	common
9.98E-05	-1.637	0.0146	common
9.47E-04	-1.51	0.0502	common
5.77E-05	-1.522	0.0106	common
1.81E-04	-1.509	0.0203	common
8.06E-04	-1.525	0.0462	common
9.86E-04	-1.542	0.0512	common
1.85E-06	-1.586	0.0014	common
8.75E-04	-1.575	0.0481	common
2.24E-06	-1.571	0.0015	common
9.82E-04	-1.61	0.0512	common
6.99E-04	-1.525	0.0428	common

2.52E-04	-1.502	0.0244	common
8.83E-04	-1.56	0.0484	common
6.67E-05	1.557	0.0115	common
1.15E-04	-1.939	0.0157	common
4.06E-05	-1.503	0.0085	common
3.27E-04	-1.508	0.028	common
1.49E-04	-1.584	0.0182	common
2.59E-05	-1.513	0.0067	common
8.15E-05	1.519	0.0128	common
1.77E-04	-1.738	0.0201	common
1.92E-04	-1.65	0.021	common
3.14E-05	-1.531	0.0075	common
5.17E-04	-1.59	0.0362	common
7.40E-05	-1.52	0.0122	common
6.82E-04	1.509	0.042	common
7.06E-05	-1.506	0.0119	common
3.87E-05	-1.655	0.0083	common
2.44E-04	-1.511	0.0239	common
2.55E-05	-1.514	0.0066	common
1.83E-05	-1.647	0.0055	common
3.30E-04	-1.727	0.0282	common
1.91E-04	-1.51	0.021	common
1.78E-04	-1.558	0.0201	common
6.99E-04	-1.659	0.0428	common
2.92E-05	-1.523	0.0072	common
7.77E-06	1.54	0.0033	common
2.77E-06	-2.111	0.0017	common
8.50E-05	1.513	0.0132	common
7.42E-04	-1.571	0.0442	common
3.11E-06	-1.945	0.0019	common
6.26E-04	-1.535	0.0402	common
6.55E-05	1.546	0.0114	common
5.36E-07	-1.513	0.0006	common
1.46E-04	-1.529	0.0179	common
5.11E-06	-1.514	0.0026	common
1.19E-05	-1.555	0.0042	common
1.60E-05	-1.634	0.0051	common
1.72E-06	-1.564	0.0013	common
9.07E-05	-1.54	0.0137	common
8.24E-04	-1.542	0.0467	common
7.71E-05	-1.55	0.0125	common
5.03E-13	1.55	0	common
1.35E-05	-1.589	0.0046	common
1.43E-04	-1.804	0.0178	common
8.02E-04	-1.619	0.0461	common
6.86E-08	1.535	0.0002	common
9.45E-06	-1.558	0.0037	common
8.64E-04	-1.531	0.0479	common

2.14E-05	-1.532	0.006	common
3.06E-05	-2.212	0.0074	common
4.20E-04	-1.53	0.0322	common
4.20E-06	-1.869	0.0023	common
5.72E-09	1.503	0	common
1.88E-04	-1.616	0.0208	common
6.07E-04	-1.642	0.0397	common
2.03E-04	-1.526	0.0217	common
2.41E-04	1.555	0.0238	common
2.00E-04	-1.533	0.0215	common
4.46E-04	-1.683	0.0333	common
2.35E-05	-1.636	0.0063	common
3.12E-05	-1.527	0.0075	common
4.84E-04	-2.016	0.0349	common
1.93E-04	-1.597	0.0211	common
1.66E-04	-1.569	0.0193	common
2.18E-04	-1.653	0.0226	common
2.46E-06	-1.573	0.0016	common
4.50E-05	2.291	0.0091	common
3.65E-04	1.502	0.0297	common
5.63E-05	-1.514	0.0104	common
7.43E-06	-1.799	0.0032	common
5.34E-04	-1.515	0.0369	common
1.17E-04	-2.35	0.0158	common
1.64E-04	-1.736	0.0192	common
5.99E-04	-1.632	0.0393	common
3.60E-07	-1.546	0.0005	common
6.01E-04	-1.662	0.0394	common
4.68E-04	-1.634	0.0342	common
2.54E-04	-2.049	0.0244	common
3.11E-05	-1.548	0.0075	common
4.28E-07	1.797	0.0006	common
7.04E-09	-1.545	0	common
5.48E-04	-1.556	0.0373	common
5.74E-04	-1.535	0.0383	common
1.58E-08	-1.624	0.0001	common
9.67E-06	-1.53	0.0038	common
3.65E-04	-1.501	0.0297	common
2.48E-04	-1.512	0.0242	common
5.46E-06	-1.505	0.0027	common
3.03E-04	-1.758	0.0269	common
9.27E-04	-1.505	0.0495	common
6.30E-04	-1.506	0.0404	common
2.03E-04	-1.502	0.0217	common
3.46E-09	1.789	0	common
8.24E-09	1.596	0	common
6.07E-05	-1.511	0.0109	common
3.80E-05	-1.845	0.0083	common

8.96E-04	-1.599	0.0487	common
3.46E-04	-1.536	0.0289	common
4.95E-04	1.604	0.0354	common
6.06E-04	-1.623	0.0396	common
2.37E-04	-1.504	0.0235	common
1.94E-08	-1.726	0.0001	common
4.80E-04	-1.51	0.0347	common
4.46E-04	-1.522	0.0333	common
2.17E-06	-1.545	0.0015	common
1.52E-05	1.527	0.0049	common
7.01E-04	-1.661	0.0428	common
7.77E-06	-1.515	0.0033	common
3.18E-04	-1.5	0.0277	common
2.79E-04	-1.521	0.0256	common
5.45E-05	-1.748	0.0102	common
2.62E-05	-1.594	0.0067	common
2.17E-10	-1.602	0	common
8.81E-04	-1.794	0.0483	common
3.97E-05	-1.6	0.0085	common
2.61E-05	-1.669	0.0067	common
2.64E-15	1.676	0	common
2.05E-09	1.544	0	common
1.31E-05	-1.525	0.0045	common
1.09E-06	-1.537	0.001	common
5.59E-04	-1.6	0.0377	common
1.78E-04	-1.514	0.0201	common
5.56E-04	1.595	0.0376	common
3.89E-05	-1.847	0.0083	common

Table S2: 70 Differentially expressed miRNAs between CF and HC

miRNA	Mean CF (log2)	Median CF (log2)	stdev CF	Mean HC (log2)	Median HC (log2)
let-7i-star	3.813	3.956	0.663	2.963	2.885
miR-1201	1.475	1.505	0.154	1.263	1.255
miR-1247	1.641	1.655	0.19	1.945	2.044
miR-1250	3.328	3.37	0.593	2.48	2.338
miR-1263	2.27	2.311	0.275	2.712	2.719
miR-1267	2.049	2.009	0.118	2.301	2.354
miR-1272	2.666	2.662	0.132	3.002	2.942
miR-1273	1.297	1.327	0.145	1.517	1.482
miR-1278	1.05	1.081	0.112	1.481	1.357
miR-128	6.577	6.619	0.314	5.843	5.884
miR-1323	2.117	2.048	0.297	2.659	2.544
miR-137	1.029	1	0.16	1.288	1.289
miR-140-5p	7.328	7.388	0.357	6.687	6.814
miR-141	1.832	1.741	0.228	1.516	1.425
miR-141-star	1.048	1.05	0.051	1.162	1.14
miR-155-star	1.207	1.264	0.202	1.494	1.477
miR-16-2-star	1.382	1.377	0.254	1.873	1.754
miR-1827	1.145	1.197	0.187	1.469	1.29
miR-184	1.157	1.21	0.177	1.475	1.451
miR-196b	2.574	2.396	0.417	1.944	1.932
miR-198	1.642	1.726	0.378	3.016	2.874
miR-19a	5.233	5.222	0.289	4.453	4.57
miR-21	6.243	6.268	0.286	5.066	5.139
miR-223-star	2.691	2.597	0.575	1.936	1.745
miR-29b-1-star	5.832	5.976	0.469	4.891	5.077
miR-29c	5.059	5.111	0.561	4.125	4.239
miR-29c-star	2.974	2.931	0.365	2.332	2.169
miR-30c-2-star	1.045	1.068	0.194	1.389	1.385
miR-30e-star	6.26	6.334	0.558	5.148	5.283
miR-320b	11.112	11.089	0.396	11.663	11.746
miR-331-3p	5.604	5.557	0.149	5.089	5.265
miR-337-3p	2.812	2.791	0.169	3.375	3.246
miR-338-5p	6.4	6.47	0.411	5.791	5.935
miR-346	3.238	3.254	0.251	2.601	2.575
miR-34a-star	1.429	1.407	0.154	1.208	1.216
miR-34c-5p	1.479	1.517	0.187	1.801	1.714
miR-367-star	1.3	1.346	0.115	1.476	1.471
miR-369-3p	1.029	1.02	0.163	1.314	1.259
miR-374a-star	2.253	2.28	0.293	2.709	2.715
miR-421	7.282	7.393	0.439	6.586	6.734
miR-450b-5p	1.119	1.092	0.146	1.594	1.556
miR-455-3p	1.723	1.721	0.397	2.565	2.431
miR-484	6.452	6.433	0.511	5.693	5.815
miR-488	1.274	1.205	0.175	1.571	1.541
miR-494	4.747	4.574	0.445	3.396	3.573

miR-503	6.329	6.313	0.344	5.603	5.797
miR-505	4.74	4.692	0.51	3.562	3.672
miR-509-3p	1.131	1.101	0.176	1.463	1.282
miR-509-5p	1.295	1.264	0.226	1.745	1.647
miR-512-5p	1.199	1.215	0.156	1.482	1.389
miR-515-5p	1.053	1.08	0.098	1.317	1.273
miR-518f-star	1.035	1.071	0.157	1.271	1.249
miR-523	2.251	2.251	0.188	1.847	1.801
miR-539	1.723	1.71	0.237	2.074	2.207
miR-548a-3p	1.264	1.241	0.216	2.53	2.402
miR-548c-3p	1.484	1.507	0.158	1.728	1.688
miR-548j	1.095	1.085	0.097	1.312	1.257
miR-548p	1.084	1.125	0.167	1.345	1.309
miR-551a	3.97	4.171	0.628	2.713	2.526
miR-551b-star	4.782	4.82	0.526	5.704	5.615
miR-570	1.167	1.192	0.124	1.697	1.635
miR-583	1.147	1.163	0.166	1.391	1.413
miR-603	1.319	1.281	0.19	1.846	1.701
miR-629	7.986	8.011	0.303	7.555	7.634
miR-630	1.13	1.159	0.066	1.356	1.351
miR-7-1-star	3.243	3.063	0.662	2.274	2.23
miR-767-5p	1.228	1.231	0.098	1.475	1.449
miR-877	4.183	4.138	0.309	4.679	4.596
miR-886-3p	1.588	1.572	0.185	2.097	2.082
miR-92b-star	4.475	4.173	0.523	3.308	3.223

Table S3: 257 unique genes showing differentially expressed isoforms between JIA and HC

Entrez ID	Gene Symbol	Isoform	MIDAS *p*-value	Mean_exon (JIA)	Median_exon (JIA)	stdev_exon (JIA)	Mean_gene (JIA)
14	AAMP	2599351	0.040911	5.616	5.495	0.428	6.214
20	ABCA2	3230661	0.035206	6.917	6.965	0.349	7.415
160	AP2A1	3838888	0.033791	9.757	9.808	0.322	8.553
160	AP2A1	3838891	0.017649	10.118	10.122	0.249	8.553
221	ALDH3B1	3337359	0.048334	6.806	6.791	0.393	7.901
301	ANXA1	3174831	0.031332	9.117	9.046	0.615	10.797
308	ANXA5	2784037	0.048343	6.839	6.848	0.859	8.687
309	ANXA6	2881754	0.032862	7.68	7.648	0.72	8.296
309	ANXA6	2881751	0.033606	7.084	6.866	0.757	8.296
327	APEH	2622220	0.047457	6.887	6.857	0.442	6.717
405	ARNT	2434676	0.037456	7.687	7.68	0.503	8.566
409	ARRB2	3707121	0.014596	11.599	11.587	0.231	11.898
472	ATM	3347684	0.049988	7.648	7.669	0.676	8.108
533	ATP6V0B	2333676	0.048477	8.922	8.925	0.331	10.398
582	BBS1	3336282	0.04845	5.944	6.057	0.412	6.291
632	BGLAP	2361427	0.022183	8.457	8.45	0.209	7.475
752	FMNL1	3723405	0.028701	9.666	9.702	0.295	10.906
836	CASP3	2796488	0.039821	7.858	7.797	0.397	7.177
841	CASP8	2522769	0.043292	10.678	10.71	0.48	9.521
967	CD63	3457179	0.018477	8.479	8.533	0.346	9.088
972	CD74	2881378	0.021344	9.609	9.557	0.709	10.465
987	LRBA	2789412	0.040965	7.521	7.628	0.842	6.119
988	CDC5L	2908591	0.04898	5.21	5.146	0.476	8.09
1021	CDK6	3061362	0.047842	6.996	7.011	0.666	6.239
1107	CHD3	3709298	0.018103	8.741	8.741	0.342	7.71
1107	CHD3	3709296	0.026456	7.913	7.895	0.371	7.71
1107	CHD3	3709295	0.027466	8.941	8.951	0.247	7.71
1107	CHD3	3709281	0.041225	8.084	8.166	0.457	7.71
1107	CHD3	3709283	0.033412	9.059	9.022	0.344	7.71
1120	CHKB	3966117	0.029697	9.135	9.176	0.316	7.968
1173	AP2M1	2655491	0.027664	11.04	11.003	0.357	8.982
1173	AP2M1	2655495	0.036043	11.708	11.715	0.322	8.982
1173	AP2M1	2655493	0.036357	10.912	10.941	0.335	8.982
1173	AP2M1	2655497	0.049672	7.372	7.406	0.351	8.982
1173	AP2M1	2655498	0.042792	7.26	7.237	0.429	8.982
1232	CCR3	2620834	0.049431	6.792	6.874	1.055	7.404
1441	CSF3R	2406789	0.04745	9.905	9.915	0.433	12.426
1488	CTBP2	3311459	0.047439	7.089	7.011	0.396	6.565
1509	CTSD	3358966	0.021596	9.689	9.676	0.334	10.617
1611	DAP	2848499	0.031052	7.736	7.694	0.306	8.56
1612	DAPK1	3177960	0.039255	6.985	6.998	0.502	8.029
1622	DBI	2502837	0.049409	7.366	7.343	0.43	6.925
1643	DDB2	3329665	0.03168	6.457	6.357	0.538	6.589
1890	TYMP	3966005	0.020135	9.897	9.994	0.562	9.158

2015	EMR1	3818617	0.036101	11.491	11.623	0.714	9.077
2034	EPAS1	2480455	0.034242	6.268	6.193	0.568	6.445
2035	EPB41	2327738	0.043028	8.366	8.429	0.403	9.641
2113	ETS1	3397597	0.037588	7.947	7.875	0.754	8.22
2131	EXT1	3150161	0.030789	7.358	7.371	0.546	6.965
2149	F2R	2816469	0.046877	4.753	4.69	0.505	5.264
2153	F5	2443383	0.039779	8.45	8.561	0.703	8.744
2733	GLE1	3190549	0.044376	8.204	8.282	0.469	7.269
2874	GPS2	3743627	0.034053	8.333	8.369	0.294	6.313
2889	RAPGEF1	3227780	0.036484	8.62	8.666	0.585	8.804
2889	RAPGEF1	3227772	0.042022	9.089	9.233	0.572	8.804
2975	GTF3C1	3686228	0.048459	7.798	7.848	0.462	7.017
3065	HDAC1	2328900	0.043795	7.582	7.582	0.35	8.721
3096	HIVEP1	2895206	0.034946	8.544	8.536	0.38	8.983
3190	HNRNPK	3212342	0.049592	7.342	7.376	0.289	10.122
3557	IL1RN	2501235	0.049068	8.621	8.598	0.642	8.864
3682	ITGAE	3741593	0.047144	7.705	7.702	0.343	5.324
3682	ITGAE	3741604	0.047478	7.94	8.038	0.572	5.324
3682	ITGAE	3741594	0.028723	7.406	7.373	0.44	5.324
3692	EIF6	3903869	0.04592	7.615	7.561	0.284	7.563
3784	KCNQ1	3317411	0.045679	8.561	8.59	0.345	7.752
3837	KPNB1	3724797	0.041966	9.969	10.079	0.437	9.574
4034	LRCH4	3064123	0.02916	8.194	8.184	0.384	9.291
4034	LRCH4	3064119	0.028326	8.87	8.855	0.367	9.291
4035	LRP1	3417911	0.048391	6.65	6.77	0.599	6.785
4297	MLL	3351435	0.024492	7.893	7.911	0.539	7.368
4297	MLL	3351436	0.030134	7.998	7.943	0.61	7.368
4318	MMP9	3887227	0.035609	10.149	10.12	0.709	10.67
4627	MYH9	3959464	0.026419	10.063	10.087	0.38	10.981
4627	MYH9	3959466	0.03442	11.044	11.109	0.33	10.981
4627	MYH9	3959463	0.020278	12.145	12.144	0.264	10.981
4627	MYH9	3959461	0.025295	12.402	12.386	0.264	10.981
4627	MYH9	3959524	0.020807	11.549	11.536	0.259	10.981
4627	MYH9	3959526	0.048271	7.743	7.732	0.452	10.981
4650	MYO9B	3824072	0.042101	9.364	9.449	0.366	9.799
4763	NF1	3717065	0.033334	7.972	7.959	0.338	7.769
4763	NF1	3717061	0.038089	7.487	7.554	0.391	7.769
4791	NFKB2	3261657	0.031954	9.629	9.637	0.387	8.746
4818	NKG7	3868999	0.049357	8.414	8.157	0.823	8.352
4851	NOTCH1	3230183	0.038594	10.213	10.165	0.312	10.167
5045	FURIN	3608423	0.021704	8.857	8.872	0.32	8.783
5311	PKD2	2735249	0.03702	6.717	6.718	0.581	5.903
5335	PLCG1	3885574	0.047059	6.869	6.72	0.627	6.906
5430	POLR2A	3708747	0.024022	8.22	8.235	0.316	9.239
5430	POLR2A	3708749	0.042134	8.325	8.331	0.413	9.239
5430	POLR2A	3708746	0.017299	9.935	9.955	0.272	9.239
5479	PPIB	3629010	0.043905	8.767	8.717	0.258	9.537
5496	PPM1G	2545813	0.046653	9.233	9.31	0.484	7.512

5496	PPM1G	2545828	0.049763	6.727	6.722	0.472	7.512
5524	PPP2R4	3190946	0.044379	5.428	5.523	0.534	5.818
5532	PPP3CB	3294531	0.018874	9.119	9.085	0.327	6.655
5566	PRKACA	3852537	0.025583	7.878	7.806	0.389	9.253
5573	PRKAR1A	3732920	0.015868	10.795	10.779	0.258	11.025
5573	PRKAR1A	3732919	0.012101	10.456	10.487	0.296	11.025
5577	PRKAR2B	3018403	0.032367	7.812	7.674	1.135	6.271
5869	RAB5B	3417179	0.030887	7.302	7.349	0.471	8.768
5887	RAD23B	3183801	0.031117	7.391	7.493	0.43	8.437
6018	RLF	2331812	0.044809	6.754	6.809	0.276	8.674
6305	SBF1	3965898	0.025448	8.536	8.54	0.342	7.243
6305	SBF1	3965896	0.025698	8.295	8.306	0.279	7.243
6389	SDHA	2798575	0.035383	6.032	6.04	0.564	8.132
6502	SKP2	2806534	0.039415	6.525	6.461	0.391	6.843
6560	SLC12A4	3696096	0.028706	8.34	8.368	0.26	7.267
6573	SLC19A1	3935023	0.01566	8.461	8.621	0.498	8.965
6602	SMARCD1	3414411	0.032476	7.049	7.045	0.25	7.76
6722	SRF	2907750	0.043881	8.275	8.219	0.319	8.957
6829	SUPT5H	3833092	0.047262	7.294	7.273	0.401	7.608
6840	SVIL	3283123	0.039744	7.476	7.576	0.324	9.374
6901	TAZ	3996373	0.03379	6.988	6.995	0.41	7.293
6901	TAZ	3996376	0.049449	8.004	8.093	0.472	7.293
6944	VPS72	2434906	0.044097	10.622	10.634	0.162	7.833
6945	MLX	3721889	0.044691	7.268	7.28	0.246	8.439
7009	TMBIM6	3414219	0.04885	9.773	9.764	0.458	11.835
7037	TFRC	2712639	0.035174	8.776	8.512	0.814	6.479
7094	TLN1	3204808	0.040014	11.328	11.354	0.21	10.417
7287	TULP1	2951565	0.03139	7.382	7.39	0.256	5.515
7391	USF1	2440524	0.046527	6.656	6.628	0.307	9.263
7391	USF1	2440535	0.044924	8.23	8.479	0.565	9.263
7462	LAT2	3008196	0.023957	8.471	8.482	0.351	8.712
7520	XRCC5	2527006	0.039496	9.973	10.026	0.394	9.643
7551	ZNF3	3063669	0.043168	6.683	6.728	0.573	6.549
7586	ZKSCAN1	3015159	0.02657	7.295	7.279	0.44	6.996
7586	ZKSCAN1	3015158	0.024653	7.442	7.476	0.411	6.996
7733	ZNF180	3864931	0.04915	4.77	4.928	0.761	4.517
7799	PRDM2	2321279	0.043973	7.451	7.488	0.39	7.335
7917	BAG6	2949167	0.03266	7.62	7.642	0.304	8.033
7917	BAG6	2949166	0.034598	8.081	8.06	0.233	8.033
7917	BAG6	2949172	0.033035	8.094	8.144	0.258	8.033
7994	KAT6A	3133173	0.040092	9.631	9.649	0.444	10.158
8209	C21orf33	3923568	0.028778	5.556	5.593	0.496	6.156
8291	DYSF	2488370	0.025067	8.555	8.628	0.398	10.555
8291	DYSF	2488368	0.024452	10.514	10.566	0.372	10.555
8291	DYSF	2488270	0.02928	8.9	8.952	0.364	10.555
8291	DYSF	2488255	0.020747	10.728	10.718	0.217	10.555
8449	DHX16	2948491	0.031263	8.485	8.476	0.322	7.311
8522	GAS7	3744977	0.030902	8.454	8.427	0.461	7.922

8556	CDC14A	2348944	0.046434	6.909	6.877	0.383	7.961
8604	SLC25A12	2586901	0.049997	5.794	5.862	0.787	5.203
8661	EIF3A	3309269	0.038842	8.02	7.947	0.4	8.325
8742	TNFSF12	3708776	0.043419	7.188	7.147	0.557	7.52
8925	HERC1	3628708	0.046025	7.953	7.909	0.476	8.518
8925	HERC1	3628658	0.041181	7.729	7.735	0.383	8.518
9057	SLC7A6	3666157	0.044889	7.621	7.558	0.62	7.554
9129	PRPF3	2358216	0.048381	6.54	6.585	0.371	9.377
9209	LRRFIP2	2669257	0.044042	7.388	7.344	0.506	7.307
9219	MTA2	3375878	0.040715	7.12	7.123	0.505	7.287
9221	NOLC1	3261510	0.037076	6.397	6.218	0.754	5.38
9296	ATP6V1F	3023220	0.042839	7.924	7.956	0.26	8.637
9632	SEC24C	3251881	0.016766	8.895	8.859	0.282	7.304
9632	SEC24C	3251879	0.011381	8.897	8.857	0.259	7.304
9632	SEC24C	3251883	0.034339	7.996	8.062	0.275	7.304
9632	SEC24C	3251856	0.035746	7.366	7.394	0.246	7.304
9632	SEC24C	3251858	0.025001	8.925	8.915	0.309	7.304
9640	ZNF592	3605858	0.022388	7.807	7.79	0.374	9.77
9663	LPIN2	3796341	0.034222	8.115	8.165	0.451	9.193
9704	DHX34	3837287	0.029101	8.341	8.343	0.282	8.324
9704	DHX34	3837290	0.023598	8.643	8.698	0.275	8.324
9748	SLK	3262437	0.048725	9.493	9.559	0.467	9.032
9772	KIAA0195	3734847	0.028831	8.568	8.548	0.326	7.307
9772	KIAA0195	3734845	0.038545	7.04	7.096	0.447	7.307
9818	NUPL1	3482261	0.040544	7.952	8.022	0.524	8.23
9826	ARHGEF11	2438677	0.047018	7.244	7.192	0.452	7.875
9826	ARHGEF11	2438672	0.042416	7.265	7.309	0.392	7.875
9826	ARHGEF11	2438664	0.034661	8.379	8.416	0.352	7.875
9855	FARP2	2536544	0.045697	5.006	5.032	0.734	5.523
9871	SEC24D	2783352	0.035487	8.341	8.33	0.35	8.481
9879	DDX46	2829522	0.043758	6.586	6.456	0.495	6.946
9879	DDX46	2829527	0.037215	8.059	8.053	0.261	6.946
9905	SGSM2	3706297	0.047511	7.34	7.322	0.415	6.918
9905	SGSM2	3706287	0.047993	6.189	6.179	0.454	6.918
9922	IQSEC1	2663436	0.039083	10.019	10.001	0.295	10.312
9922	IQSEC1	2663435	0.035752	8.865	8.879	0.34	10.312
9968	MED12	3980763	0.04907	8.286	8.36	0.396	9.358
10014	HDAC5	3758854	0.033581	7.24	7.233	0.343	8.375
10036	CHAF1A	3817530	0.046422	6.154	6.144	0.376	5.591
10075	HUWE1	4009343	0.046905	7.868	7.862	0.378	7.958
10106	CTDSP2	3458922	0.041447	10.025	9.988	0.356	10.889
10159	ATP6AP2	3974574	0.042885	6.54	6.639	0.448	9.629
10178	ODZ1	4020726	0.036286	6.379	6.541	0.966	5.673
10197	PSME3	3722181	0.030143	9.752	9.743	0.335	9.899
10211	FLOT1	2948620	0.044021	9.291	9.313	0.43	9.398
10238	DCAF7	3730755	0.019063	9.626	9.599	0.27	8.356
10295	BCKDK	3656850	0.04987	8.094	8.109	0.289	8.165
10295	BCKDK	3656849	0.0427	8.483	8.55	0.28	8.165

10299	MARCH6	2801682	0.023147	7.726	7.74	0.325	9.89
10362	HMG20B	3817057	0.046585	6.199	6.241	0.559	7.325
10444	ZER1	3226682	0.027862	7.249	7.278	0.35	8.062
10472	ZNF238	2388812	0.04452	8.34	8.333	0.298	9.297
10526	IPO8	3449350	0.048159	6.795	6.85	0.415	6.666
10540	DCTN2	3458633	0.045785	7.084	7.215	0.568	7.55
10641	NPRL2	2675275	0.037864	7.126	7.084	0.329	6.928
10765	KDM5B	2451364	0.039398	8.654	8.682	0.394	8.097
10788	IQGAP2	2816405	0.027781	8.754	8.916	0.548	9.206
10788	IQGAP2	2816301	0.039339	9.988	10.046	0.378	9.206
10788	IQGAP2	2816347	0.046469	10.766	10.753	0.401	9.206
10788	IQGAP2	2816299	0.030965	10.864	10.977	0.426	9.206
10847	SRCAP	3656496	0.037084	9.055	9.002	0.352	8.55
10847	SRCAP	3656474	0.033465	7.865	7.905	0.323	8.55
10847	SRCAP	3656473	0.026083	7.649	7.648	0.258	8.55
10847	SRCAP	3656505	0.032294	6.751	6.7	0.374	8.55
10885	WDR3	2354095	0.037583	6.419	6.309	0.539	4.604
10905	MAN1A2	2353904	0.017277	8.656	8.722	0.573	6.476
10905	MAN1A2	2353901	0.037508	7.999	8.005	0.536	6.476
10905	MAN1A2	2353910	0.027551	8.17	8.141	0.457	6.476
10905	MAN1A2	2353899	0.040846	8.44	8.376	0.417	6.476
10922	FASTK	3079359	0.031106	7.724	7.706	0.278	6.808
10970	CKAP4	3469691	0.042259	8.706	8.69	0.468	8.98
11000	SLC27A3	2359887	0.033669	7.183	7.294	0.528	7.232
11000	SLC27A3	2359889	0.034527	7.822	7.811	0.33	7.232
11012	KLK11	3868849	0.036152	8.015	8.005	0.325	4.956
11027	LILRA2	3841409	0.03632	8.963	9.031	0.372	10.295
11035	RIPK3	3558245	0.035574	7.662	7.635	0.413	7.498
11176	BAZ2A	3458020	0.026452	10.437	10.524	0.276	10.119
11190	CEP250	3883355	0.046447	3.63	3.662	0.546	4.931
11214	AKAP13	3606481	0.029613	10.235	10.205	0.369	9.512
11338	U2AF2	3842360	0.029587	8.806	8.787	0.34	8.165
22823	MTF2	2346973	0.028218	7.949	7.935	0.467	7.404
22853	LMTK2	3014203	0.026647	8.847	8.893	0.306	8.891
22856	CHSY1	3642090	0.031221	10.304	10.326	0.487	10.158
22870	PPP6R1	3871292	0.034076	10.694	10.683	0.318	8.975
22878	TRAPPC8	3803247	0.032721	7.503	7.636	0.447	7.783
22897	CEP164	3350926	0.03888	6.398	6.514	0.572	6.142
22904	SBNO2	3845006	0.045202	7.815	7.933	0.395	9.034
22906	TRAK1	2619187	0.034233	7.791	7.734	0.367	7.337
22980	TCF25	3674477	0.019823	8.908	8.916	0.35	8.17
23013	SPEN	2322139	0.012992	11.496	11.511	0.163	9.317
23038	WDTC1	2326938	0.013197	9.498	9.567	0.313	9.148
23038	WDTC1	2326937	0.014297	9.196	9.228	0.258	9.148
23038	WDTC1	2326936	0.038997	7.018	7.053	0.38	9.148
23152	CIC	3834728	0.04482	10.52	10.536	0.322	7.408
23152	CIC	3834713	0.032646	7.324	7.284	0.452	7.408
23162	MAPK8IP3	3644130	0.035131	6.42	6.442	0.416	7.292

Table S7. Primers used for quantitative real-time PCR validation

Gene symbol	Primer direction	Sequence(5'~3')
ANXA3	Forward	GGCAGCTGATTGTTAAGGAATATC
	Reverse	CCTGCTTGTCCTGGTAGTTAAG
CALR	Forward	TCTACGGTGACGAGGAGAAA
	Reverse	CATGTCTGTCTGGTCCAAACTAT
CD22	Forward	TGTTACCCGGTATGAATGGAAA
	Reverse	CCAGAGTGAATCTCGGAAAGG
CDK5RAP2	Forward	CAGGCCAGTGATGTGGATTA
	Reverse	CTCAGGAGATTCAGGCAGAAG
CEP250	Forward	AACACCCTGAAGACAGAAGTAG
	Reverse	CGACCTGCAAAGCATTTCTC
CST7	Forward	GCAGCCAGATACAGTGTTGA
	Reverse	AAGGTGTGGTTGGTTTGGA
DCUN1D1	Forward	CCATGAACTCCTGTTTGGAGA
	Reverse	AGGATCATTGGACAGGAAGAAG
RBMS1	Forward	GTGGTACAAGTCGTGGTGTT
	Reverse	CTTCTCTATGCCATGGTCTTCC
HLA-DRB1	Forward	GAACGGCCAGGAAGAGAAG
	Reverse	CGACTCCACTCAGCATCTTG
IL8	Forward	TTGGCAGCCTTCCTGATTT
	Reverse	AGACAGAGCTCTCTTCCATCA
MMP25	Forward	GGCAGCGTGTGGAAGAA
	Reverse	GGCAAAGTCGATGAGGATGT
PLK1S1	Forward	TCCATCTTCTCCCTCCATCTT
	Reverse	TTCTCCTTGGGAAGGTGTTTC
PLXNB2	Forward	GTGGATGCGGTACAGAAGAA
	Reverse	CGGTACACCTGGTCAATGAT
PRKACA	Forward	TTCCGTTCCTCGTCAAACTC
	Reverse	GATCCAGCGAGTGCAGATAC
RPL14	Forward	TGATCAGAACAGGGCTTTGG
	Reverse	TCTCTGGCTTCAATCTTCTTGG
S100A10	Forward	CTTAACAAAGGAGGACCTGAGAG
	Reverse	CCCTTCTGCTTCATGTGTACTA
SCARF2	Forward	GCGCCACCGGTTTCTAT
	Reverse	GACTGGAAGTCGCAGTGTC
SGK1	Forward	TGCATTCACTGAACATCGTTTATAG
	Reverse	CAGTCCACAGTCCTGTCATAAG
TBC1D15	Forward	AGCAGAATGGGACATGGTTAATA
	Reverse	GAAAGTGTAGAGCAGGGAGAAC
TMEM123	Forward	CCAGTGCTAACTCAACAGAGAC
	Reverse	GGTGTAGACTTTAAGGTGGTAGAAG
VCAN	Forward	CAGCTCTTTGCTGCCTATGA
	Reverse	ACCATCCAGATGATCCACATAAC

Table S8. The target sequences of miRNAs for qRT-PCR

Name on Array	Assay Name	Assay ID
hsa-miR-34a	hsa-miR-34a	426
hsa-miR-127-3p	hsa-miR-127	452
hsa-miR-409-3p	hsa-miR-409-3p	2332
hsa-miR-494	hsa-miR-494	2365
hsa-miR-551a	hsa-miR-551a	1519
hsa-miR-933	hsa-miR-933	2176
hsa-miR-379	mmu-miR-379	1138
has-miR-19a	hsa-miR-19a	395
hsa-miR-128	hsa-miR-128a	2216
hsa-miR-140-5p	mmu-miR-140	1187
hsa-miR-21	hsa-miR-21	397
hsa-miR-29b-1*	hsa-miR-29b-1*	2165
hsa-miR-421	hsa-miR-421	2700
hsa-miR-484	hsa-miR-484	1821
hsa-miR-503	hsa-miR-503	1048
